# Domain-specific expression of meristematic genes is defined by the LITTLE ZIPPER protein DTM in tomato

**DOI:** 10.1038/s42003-019-0368-8

**Published:** 2019-04-23

**Authors:** Qian Xu, Rong Li, Lin Weng, Yuan Sun, Meng Li, Han Xiao

**Affiliations:** National Key Laboratory of Plant Molecular Genetics, CAS Centre for Excellence in Molecular Plant Sciences, Institute of Plant Physiology and Ecology, Chinese Academy of Sciences, 300 Fenglin Rd, 200032 Shanghai, China

**Keywords:** Shoot apical meristem, Plant signalling

## Abstract

Shoot meristems, which harbor a small population of stem cells, are responsible for generating new above-ground organs in plants. The proliferation and differentiation of these stem cells is regulated by a genetic pathway involving two key meristematic genes: *CLAVATA3* (*CLV3*) and *WUSCHEL* (*WUS*). However, it is not well understood how *CLV3* and *WUS* expression domains in the shoot meristems are specified and maintained during post-embryogenic development. Here, we show that a tomato mutant with fasciated stems, flowers and fruits, due to impaired stem cell activity, is defective in a LITTLE ZIPPER gene denoted as *DEFECTIVE TOMATO MERISTEM* (*DTM*). DTM forms a negative feedback loop with class III homeodomain-leucine zipper (HD-ZIP III) transcription factors to confine *CLV3* and *WUS* expression to specific domains of the shoot meristems. Our findings reveal a new layer of complexity in the regulation of plant stem cell homeostasis.

## Abstract (web summary)

Qian Xu et al. show that shoot apical meristem activity in tomato is maintained by a LITTLE ZIPPER protein known as DTM. This protein negatively regulates a class of HD-ZIP III transcription factors, ultimately maintaining stem cell homeostasis.

## Introduction

Shoot apical meristems (SAMs) provide meristematic cells for the formation of plant aerial organs, and maintaining SAM activity is crucial for plants to complete their life cycles and to adapt to changing environments^1^. Within the SAM, stem cell niches are maintained by the CLAVATA-WUSHEL (CLV-WUS) feedback loop^2–5^. In this regulatory module, WUS controls stem cell fate by preventing cell differentiation, while CLV3 regulates cell division to restrict SAM size^6,7^. At the transcription level, CLV3 restricts *WUS* expression to a limited number of cells, while low WUS activity promotes *CLV3* expression^8,9^. In addition, *SHOOTMERISTEMLESS* (*STM*) prevents the entrance of stem cell differentiation in the SAM^10–13^. It has been well documented that the coordinated actions of these three meristematic genes in maintaining the SAM are highly dependent on their layer-specific and overlapping expression^2,4,14^.

Class III homeodomain-leucine zipper (HD-ZIP III) genes regulate SAM activity in addition to the identities of the adaxial tissues of lateral organs in *Arabidopsis thaliana*^15,16^. The *A. thaliana* loss-of-function HD-ZIP III triple mutant, *revoluta phabulosa phavoluta* (*rev phb phv*), only forms pin-like shoots, whereas high expression of HD-ZIP III genes causes enlarged SAMs^17–19^. Recently, it was shown that HD-ZIP III proteins regulate *WUS* expression through interaction with A-type ARABIDOPSIS RESPONSE REGULATOR proteins during de novo shoot regeneration^20^. Furthermore, REV directly activates *STM* expression in the meristematic cells at the leaf axil for axillary meristem formation^21^. However, regulation of SAM activity by HD-ZIP III transcription factors can be WUS- dependent or independent^19,22^, suggesting the involvement of additional modifiers.

HD-ZIP III transcription factors are regulated at the transcriptional, post-transcriptional, and post-translational levels^23–27^. For example, LITTLE ZIPPER proteins (ZPRs) inhibit HD-ZIP III activities by forming presumably nonfunctional heterodimers with them^25,27^. Thus, ZPRs control SAM development through suppression of HD-ZIP III activities. The *A. thaliana* double mutant of *ZPR3* and *ZPR4*, *zpr3–2 zpr4–2*, produces ectopic shoot meristems, while overexpression of *ZPR3* causes early meristem termination^25,27^.

The genetic program governing the SAM stem cell system is generally conserved across plant species^2^. Changes in meristem size have also been observed in the tomato (*Solanum lycopersicum*) *locule number* and *fasciated* (*fas*) mutants, which have mutations in the *WUS* and *CLV3* orthologs *SlWUS* and *SlCLV3*, respectively^28,29^. However, unlike *A. thaliana*, tomato is a typical sympodial plant: its shoot development is indeterminate since after primary shoot meristems terminate into inflorescences, sympodial meristems (SYM) are formed at the leaf axil immediately beneath the inflorescence meristems to sustain its growth^14^. The difference in SAM activity between the two species is likely due to diversified regulatory mechanisms, as exemplified by SAM doming being regulated by *LATE TERMINATION*, which occurs in tomato but is absent in *A. thaliana*^30^. To identify new genetic components that regulate SAM activity in tomato, we screened for defective meristem mutants in a large ethyl methanesulfonate mutagenized tomato population. Characterization of one such mutant revealed that a LITTLE ZIPPER protein DTM regulates SAM activity through its negative post-translational regulation of SlREV, the tomato ortholog of REV. Mutations in *DTM* and *SlREV* cause mutually exclusive changes in expression patterns of the meristematic genes *SlCLV3* and *SlWUS* in the SAM. We propose that a DTM-SlREV feedback loop defines the domain-specific action of CLV-WUS signaling, thereby maintaining stem cell homeostasis during post-embryogenic development.

## Results

### The *DTM* gene encodes a LITTLE ZIPPER protein

**Fig. 1:**
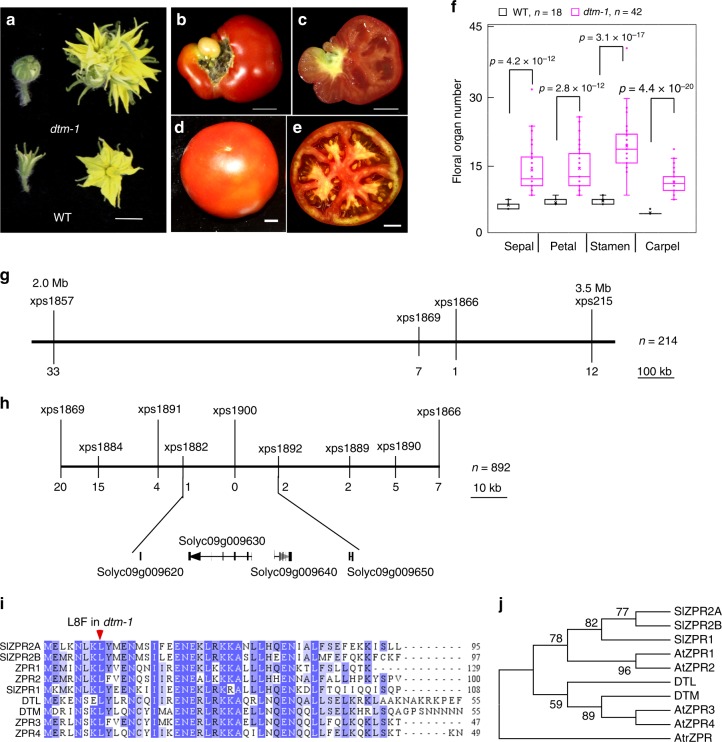
Isolation of the *DTM* gene. **a**–**e** images of flower buds and flowers at anthesis (**a**) and fruits (**b**–**e**) from *dtm-1* (**a**–**c**) and wild type (**a**, **d**, and **e**) plants in the LA2397 background. Images of longitudinally sectioned *dtm-1* fruits (**c**) showed that no seeds formed inside, compared with seeded wild type fruits (**e**). Scale bars, 1 cm. **f** floral organ number of *dtm-1* and wild type. Data were collected from flowers at anthesis from 10 plants per genotype. The boxplots show the first quartile and third quartiles, split by the median. Means are indicated by an X in each box. A Welch’s *t*-test was applied to compare the differences in means between *dtm-1* and wild type. **g** low resolution mapping of the *DTM* locus. *DTM* was mapped to a 1.5 Mb interval using 214 *dtm-1* plants from a *S. pimpinellifolium* LA1781 × *dtm-1* F_2_ population. **h** fine mapping of *DTM*. The *DTM* locus was further narrowed down to a 25.3 kb region between the xps1882 and xps1892 markers using a total of 892 mutants. The numbers under the lines marking the genetic markers are the numbers of recombinants. **i** protein sequence alignment of DTM and its homologs in tomato and *Arabidopsis thaliana*. The missense mutation identified in *dtm-1* is indicated by a red arrowhead. **j** maximum parsimony tree of DTM and its homologs in tomato and *A. thaliana*. The consensus phylogenetic tree was constructed using MEGA7.0 with 1000 bootstrap replications and the tree was rooted by an outgroup ZPR protein, AtrZPR (NCBI accession: XP 006851800.1), from *Amborella trichopoda*. The numbers next to branches show the percentages of the replicate trees (only 50% or higher reported) in the bootstrap test

The SAMs of plants provide stem cells for the formation of aerial organs, such as flowers and fruits, which are often associated with the target traits of crop breeding. Through screening of an ethyl methanesulfonate mutagenized population of the cultivated tomato LA2397 for mutations affecting flower and fruit development, we identified a meristem defect mutant, which we named *defective tomato meristems-1* (*dtm-1*). This mutant was first noted for its enlarged fasciated flowers and increased numbers of floral organ (Fig. 1a–f). *dtm-1* has seedless fruits, which we determined was due to female sterility, since the mutation was transmitted to wild type plants by pollinating with samples of *dtm-1* pollens. To identify the causal mutation underlying the mutant phenotype, *dtm-1* was crossed as the pollen donor to *Solanum pimpinellifolium* LA1781, an accession of a wild relative of cultivated tomato, to generate a F_2_ mapping population. Using 214 *dtm-1* plants, *DTM* was mapped to a 1.5 Mb interval (SL2.50ch09:2.0–3.5 Mb) between markers xps1857 and xps215 (Fig. 1g). Fine mapping, using an additional 678 *dtm-1* plants and newly developed markers, further narrowed down the *DTM* locus to a 25.3 kb region between the xps1882 and xps1892 markers (Fig. 1h). Based on the ITAG2.4 annotation from the sol genomics network database (SGN, https://solgenomics.net/), this region was determined to contain four protein encoding sequences, but no mutation was found in any of them in *dtm-1*. We then performed ab initio gene prediction with the 25.3 kb genomic sequences using FGENESH (www.softberry.com), and found that *Solyc09g009620* was annotated incorrectly, missing a noncoding exon and 45 nucleotides (15 amino acids) at its 5′-end. This was confirmed by rapid amplification of cDNA ends analysis (Supplementary Fig. 1). After resequencing the corrected *Solyc09g009620* coding sequence, we found that *dtm-1* has a single point mutation near the beginning of the second exon: a C to T transversion (C22T) that causes the conserved leucine at position 8 to be changed to phenylalanine (L8F) (Fig. 1i).

DTM shares high amino acid sequence similarity with the *A. thaliana* ZPR4 protein (77% identity, 35/47), a member of a small gene family that is widely present in land plants^25,27^. The tomato genome encodes four additional ZPR proteins, named DTM-like (DTL, Solyc11g007100), SlZPR1 (Solyc01g091490), SlZPR2A (Solyc08g007570), and SlZPR2B (Solyc08g079690) based on their homology with *A. thaliana* ZPR proteins (Fig. 1j). In a phylogenetic analysis, DTM and its closest homolog DTL (69% identity) grouped with *A. thaliana* ZPR3 and ZPR4, while the other three tomato ZPR proteins were more similar to *A. thaliana* ZPR1 and ZPR2 (Fig. 1j).

### *dtm-1* exhibits multiple meristem defects

**Fig. 2:**
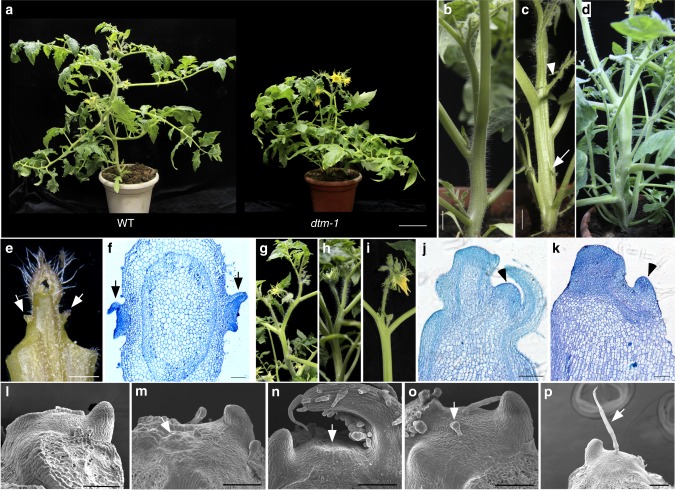
Phenotypes of the *dtm-1* mutant and wild type. **a** images of representative adult *dtm-1* and wild type plants. Scale bar, 10 cm. **b**–**d** images of main stems showing axillary bud development in wild type (LA2397, **b**) and *dtm-1* (**c**, **d**). The arrowhead and arrow in (**c**) indicate a side shoot and a leafy structure ectopically formed between two consecutive leaves rather than at the leaf axils. Scale bars, 1 cm. **e** a dissected shoot apex from *dtm-1* plants showing two symmetrical buds (indicated by arrows) formed on the stem. Scale bar, 100 μm. **f** cross section of a *dtm-1* primary stem showing two axillary meristems (indicated by arrows) formed almost simultaneously at opposite positions. Scale bar, 100 μm. **g**–**i** images of primary (**g**, **h**) and side (**i**) shoots of wild type (**g**) and *dtm-1* plants (**h**, **i**). **j**–**k** longitudinal sections of a wild type (**j**) and a *dtm-1* (**k**) shoot apex at 15 days after germination (DAG). Arrowheads indicate sympodial meristems (SYMs). **l**–**p** scanning electron microscopy (SEM) images of shoot apical meristems (SAMs) from wild type (**l**) and *dtm-1* (**m**–**p**) seedlings. The *dtm-1* SAMs had a rough surface (**m**), doming defect (**n**), and produced precocious leaf primordia (**o**) and ectopic trichomes (**o**, **p**) indicated by arrows. Scale bars, 100 μm

Although *dtm-1* plants looked similar to wild type at the seedling stage, close examination revealed that the mutant had defects in axillary shoot formation. Specifically, axillary buds were formed above the leaf axils, rather than at the leaf axils, as in wild type (Fig. 2a–d). Occasionally, leafy organs, instead of axillary buds, were formed on the stems between two consecutive leaves (Fig. 2c). Additionally, two lateral shoots were often observed on opposite sides of the stems, and anatomical analysis of cross-sections of the upper shoots revealed that two axillary meristems formed simultaneously (Fig. 2e, f). These axillary buds were able to develop into functional shoots with fasciated flowers like those on the primary shoots (Fig. 2g–i). However, all branches terminated in single inflorescences, indicating that SYM development on the branches was defective (Fig. 2i). Compared with wild type, SYM formation on primary shoots was slightly delayed in the *dtm-1* mutant (Fig. 2j, k). These observations suggest that SAM activity is not properly maintained in the *dtm-1* mutant.

By dissecting the shoot apices, we found that *dtm-1* SAMs were flatter and wider than those of wild type, as revealed by scanning electron microscopy (SEM) (Fig. 2l, m). In addition, the dome surface of *dtm-1* SAMs appeared wrinkled, and ectopic trichomes often formed on the epidermis (Fig. 2n–p). As in many other plant species, the SAM gradually develops into a dome structure in tomato^30^. To better understand the timing of *DTM* action on SAM development, we analyzed the doming of *dtm-1* SAMs. At 3 days after germination (DAG), we observed no difference in SAM morphology between *dtm-1* and wild type, except that the SAMs of the mutant were wider (Supplementary Fig. 2). However, doming was limited in *dtm-1* and its SAMs remained flat or slightly bulged until the developmental transition to inflorescence meristems.

Interestingly, in the F_2_ population derived from a cross between *dtm-1* and LA1781, we observed extremely elongated flower stalks and a wide range of variations in fruit fasciation (Supplementary Fig. 3). This suggests that the *dtm-1* mutation has pleiotropic effects on flower and fruit development due to undetermined genetic modifiers in the LA1781 background, or to recombination events. To test whether the mutation has different extents of phenotypic penetration in different genetic backgrounds, we also introduced the *dtm-1* allele into *S. lycopersicum* cv. Moneymaker, a cultivar that has been widely used for genetic and molecular studies^31^, and into the wild species accession LA1781. In the three genetic backgrounds, the *dtm-1* mutation had very similar, if not identical, effects on SAM development. All mutant phenotypes observed in the LA2397 background were observed in both the LA1781 and Moneymaker backgrounds (Supplementary Fig. 4).

### Phenotypes of null *dtm* alleles created by CRISPR-Cas9

**Fig. 3:**
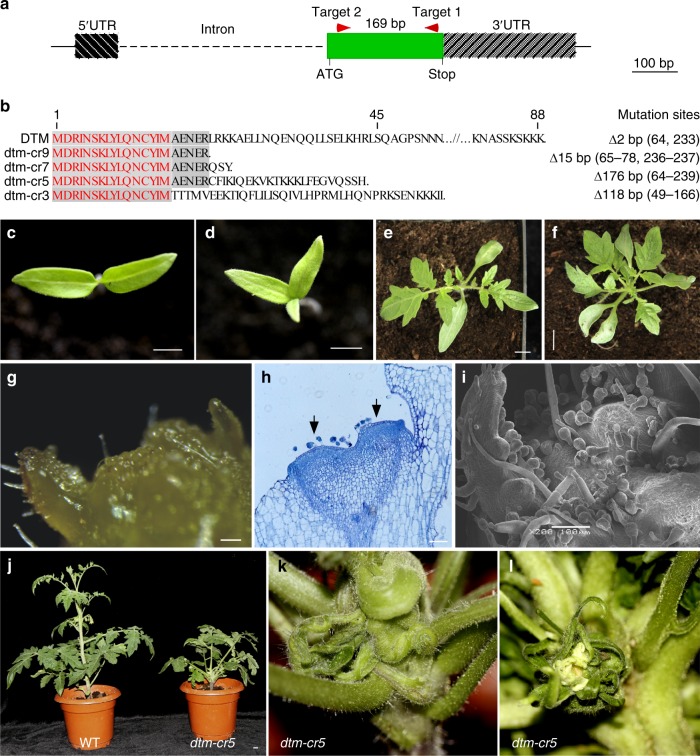
Phenotypes of the loss-of-function *dtm-cr5* alleles created by CRISPR-Cas9. **a** schematic illustration of the gRNA target design aiming for large deletions in the coding region of *DTM*. The two single gRNA target sites are indicated by two red arrowheads. **b** alignment of translated protein sequences from the four *dtm-cr* alleles containing putative loss-of-function mutations. Sequences identical to wild type DTM are shaded and the first 15 amino acids not changed by mutations in the four alleles are highlighted in red. The nature of the mutations in each of the four alleles is indicated on the right. **c**–**f** representative images of *dtm-cr5* (**d**, **f**) and wild type (Moneymaker, **c**, **e**) seedlings at 3 and 9 days after germination (DAG). The four mutant alleles had identical phenotypes including tricot formation (**d**) and precocious leaf formation (**f**). Only *dtm-cr5* images are shown. **g**–**i** dissected *dtm-cr5* apices showing shoot apical meristem (SAM)developmental abnormality as revealed by stereomicroscopy (**g**), paraffin sectioning (**h**), and scanning electron microscopy (SEM) (**i**). The *dtm-cr5* apex was flat with a very rough surface (**g**) and two SAM-like structures (indicated by arrows) were observed in longitudinal sections (**h**). The SEM image (**i**) shows dense trichomes formed on a *dtm-cr5* SAM. **j** images of *dtm-cr5* and wild type adult plants. **k**–**l** two close-up images showing an extremely fasciated stem (**k**) and flower (**l**) from the *dtm-cr5* plant shown in (**j**). Scale bars, 5 cm (**c**, **d**), 1 cm (**e**, **f**, **j**) and 100 μm (**g**–**i**)

Since the phenotypic abnormality observed in the *dtm-1* mutant is caused by a missense mutation, to gain insight into its loss-of-function effects on SAM development, we generated several null alleles of the *DTM* gene by clustered regularly interspaced short palindromic repeats (CRISPR)-Cas9 genome editing, using two single guide sequences located 169 bp apart on the second exon (Fig. 3a). In total, we obtained four lines with 2–176 bp deletions in the protein coding region that likely disrupted *DTM* functionality, from more than ten primary transformants (Fig. 3b). The four loss-of-function alleles had the same phenotypes and we chose line *dtm-cr5*, which has a 176 bp deletion (nucleotide 64–239), for further analysis.

Unlike the *dtm-1* allele, *dtm-cr5* seedlings had abnormal cotyledon numbers, with almost all of the mutants (104 out of 107) having more than two (Fig. 3c–f). Examination of the *dtm-cr5* apices revealed that its SAM development was more severely perturbed than in *dtm-1*. Domed SAM structures were rarely present in the *dtm-cr5* seedlings at 3–12 DAG, although multiple SAM-like structures were identified in longitudinal sections of their shoot apices (Fig. 3g, h; Supplementary Fig. 5). Moreover, *dtm-cr5* apices were densely covered by trichomes (Fig. 3i). Later in development, the adult *dtm-cr5* plants were dwarfed, with extremely fasciated and twisted stems, clustered leaves (Fig. 3j, k), and occasionally flowers formed without petals (Fig. 3l).

Since *SlCLV3* is also involved in SAM doming, we tested for genetic interactions between *SlCLV3* and *DTM* by crossing *dtm-1* with *S. lycopersicum* cv. Super Beefsteak, which has a chromosomal conversion that downregulates *SlCLV3* expression^29^. The double mutant, *dtm-1 fas*, was indistinguishable from *dtm-1* in SAM morphology: both produced flat SAMs with ectopic trichomes on the epidermis (Supplementary Fig. 6). However, SYM development was abolished in *dtm-1 fas* mutants and side shoots were replaced by inflorescence-like structures (Supplementary Fig. 6), indicating that the two genes may have additive effects on lateral meristem development.

### Interactions between DTM and HD-ZIP III proteins

**Fig. 4:**
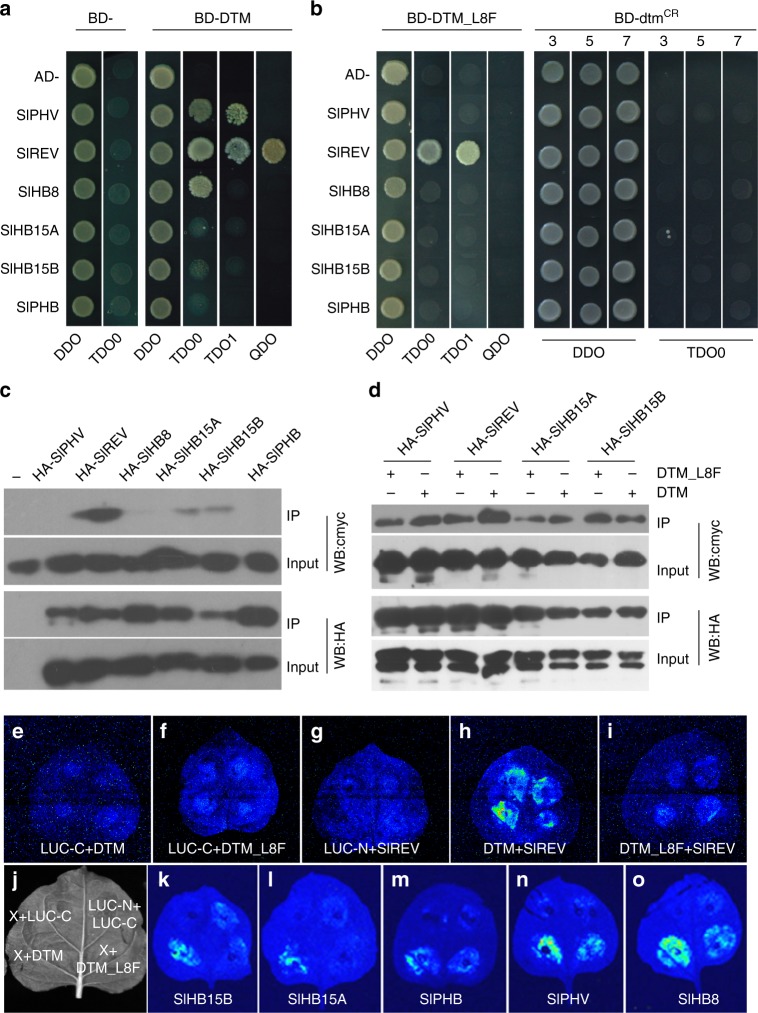
The L8F mutation in DTM weakens the interactions between DTM and tomato HD-ZIP III proteins. **a** interactions between DTM and tomato HD-ZIP III proteins SlREV, SlPHB, SlPHV, SlHB8, SlHB15A, and SlHB15B in yeast cells. *DTM* cDNA was fused in frame to the coding sequence of the GAL4 DNA binding domain (BD), and the full-length cDNA of the six HD-ZIP III genes was fused to the coding sequence of the GAL4 activation domain (AD). The numbers above the images represent cell densities obtained by 0, 1-, 10-, and 100-times dilutions from yeast cell suspensions at an OD_600_ of 1.0. **b** interactions between DTM mutants and the six tomato HD-ZIP III proteins in yeast cells. The cDNA encoding DTM_L8F, dtm^cr3^, dtm^cr5^, and dtm^cr7^ indicated by the numbers above the images were PCR amplified from *dtm-1*, *dtm-cr3*, *dtm-cr5* and *dtm-cr7* DNA. **c** interactions between DTM and the six tomato HD-ZIP III proteins tested by a pulldown assay. **d** comparison of binding ability between DTM and DTM_L8F to the HD-ZIP III proteins SlREV, SlPHV, SlHB15A, and SlHB15B. Pulldown assays were conducted using *Escherichia coli* expressed proteins either fused to the cmyc (DTM and DTM_L8F) or the HA (HD-ZIP IIIs) tag. Protein complexes were immunoprecipitated using an anti-HA antibody (**c**, **d**). **e**–**o** interactions between DTM and the mutant form DTM_L8F and HD-ZIP III proteins in tobacco leaves. DTM (or DTM_L8F), and the six tomato HD-ZIP III proteins were fused to the N- and C-terminal portion of LUC, respectively. Constructs expressing two terminal halves of LUC only, or one terminal half of LUC not fused with a target protein in combination with another half fused with the target to be tested, served as negative controls. The white field image (**j**) depicts the experiment setup, in which ‘X’ represents a specific HD-ZIP III member to be tested as shown under each leaf in (**k**–**o**). DDO, SD/-Leu-Trp; TDO0 and TDO1, SD/-Leu-Trp-His with 0 and 1 mM 3-amino-1,2,4- triazole (3-AT), respectively; QDO, SD/-Leu-Trp-His-Ade. IP, immunoprecipitation; WB, Western blot

LITTLE ZIPPER proteins function as post-translational suppressors of class III HD-ZIP transcription factors by inhibiting their homodimerization^25,27^. In tomato, there are six HD-ZIP III members, of which Solyc11g069470 has been named SlREV, based on its sequence similarity to *A. thaliana* class III HD-ZIP transcription factors^32^. We assigned the following names to the remaining tomato HD-ZIP III proteins based on sequence similarity and phylogenetic analysis: SlPHB (Solyc02g024070), SlPHV (Solyc02g069830), SlHB15A (Solyc03g120910), SlHB15B (Solyc12g044410), and SlHB8 (Solyc08g066500) (Supplementary Fig. 7). We tested the interactions between DTM and the tomato HD-ZIP III proteins by yeast two hybrid and pulldown assays. In yeast, DTM interacted with full-length SlREV, SlPHV and SlHB8 (Fig. 4a), and with the individual N-terminal regions, which each contains a homeodomain and a leucine zipper domain, of all the tomato HD-ZIP III members except SlHB15A (Supplementary Fig. 8a). Using *Escherichia coli* expressed proteins, cmyc-DTM was immunoprecipitated with an HA antibody when incubated with protein extractions containing HA-tagged SlPHV, SlREV, SlHB8, SlHB15A or SlHB15B, but not HA-SlPHB (Fig. 4c). This suggests that DTM may interact with several members of the tomato HD-ZIP III transcription factor family.

The *dtm-1* mutant has a missense mutation (L8F) at the interhelical interface in the second heptad of the leucine zipper domain, which presumably affects its interaction with the HD-ZIP III proteins^25^. Indeed, compared to wild type DTM, the mutant form DTM_L8F and the three truncated proteins created by CRISPR-Cas9 showed weaker and no interaction, respectively, with HD-ZIP III members in yeast (Fig. 4b; Supplementary Fig. 8a). The weakened interaction between DTM_L8F and full-length or the N-terminal regions of HD-ZIP III proteins was further confirmed by pulldown assays (Fig. 4d; Supplementary Fig. 8b, c). To investigate the interactions between DTM and the HD-ZIP III proteins in vivo, we conducted a biomolecular fluorescence complementation assay in tobacco leaves^33^. DTM or DTM_L8F were separately heterologously expressed in tobacco leaves as translational fusions with the N-terminal half of the firefly luciferase (LUC) reporter protein, together with the C-terminus of LUC fused with different HD-ZIP III proteins. We observed that wild type DTM interacted with all six tomato HD-ZIP III proteins, while its mutant form, DTM_L8F, showed much weaker interactions (Fig. 4e–o).

**Fig. 5:**
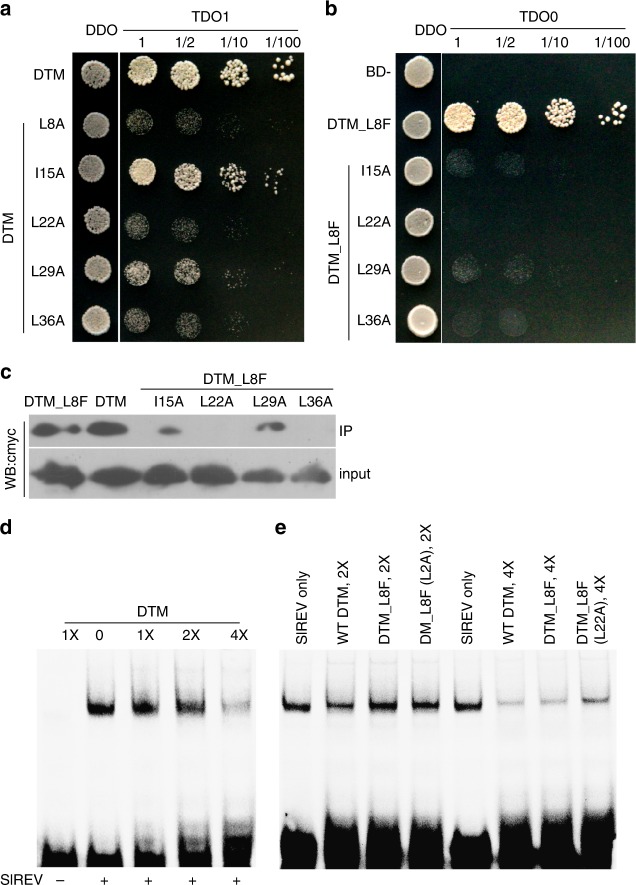
Mutagenesis of the DTM protein. **a** interactions between SlREV (fused to the activation domain, AD) and five DTM mutants with single mutations in the conserved Leu or Ile residues in each heptad (fused to the binding domain, BD) in yeast. The five conserved Leu or Ile residues were individually mutated to Ala. **b** interactions between SlREV and four DTM double mutants derived from DTM_L8F in yeast. **c** binding activities of DTM and its mutant forms to its partner, SlREV. DTM and its mutant forms were fused to cmyc and their binding affinities to SlREV (fused to HA) were assayed by pulldown using an anti-HA antibody. **d** electrophoretic mobility shift assay showing dosage-dependent inhibition of DTM on SlREV binding ability to its target sequence. **e** inhibitive effect of single and double mutations in the conserved residues of DTM on SlREV binding ability to its target sequence. Both SlREV (1–264 aa) and DTM were synthesized using wheat germ extract, and different concentrations of DTM (**d**) and/or its mutated forms (**e**) were tested for their inhibitive effects on SlREV binding to a Cy5-labeled HB9 DNA duplex. 3-AT, 3-amino-1,2,4- triazole; IP, immunoprecipitation; WB, Western blot

When the conserved Leu or Ile residues in the five heptads (2nd–6th) of the leucine zipper domain in DTM were individually mutated to Ala, its interaction with SlREV in yeast was weakened (Fig. 5a). However, mutation of the Ile in the third heptad caused only a slightly attenuation of the interaction between DTM_I15A and SlREV, suggesting that it is less important for the DTM-SlREV interaction. Mutagenesis of DTM_L8F further confirmed the weak effect of the I15A mutation as we still observed an interaction between DTM_L8F(I15A) and SlREV using both the yeast two hybrid and pulldown assays (Fig. 5b, c). DTM_L8F(L29A) interacted with SlREV in pulldown assays, but not in yeast two hybrid assays. No interaction was found between DTM_L8F(L22A), DTM_L8F(L36A) and SlREV. The mutagenesis analysis suggests that the leucine residues in heptad two, four, and six are critical for the interaction between DTM and HD-ZIP III transcription factors.

Previous studies have shown that LITTLE ZIPPER proteins inhibit DNA binding of HD-ZIP III transcription factors^25,27^. We confirmed that DTM inhibited SlREV binding to the HB9 duplex containing consensus sequence for REV using an electrophoretic mobility shift assay, and further showed that the inhibition by DTM was dosage-dependent (Fig. 5d, e). Moreover, consistent with the weakened interactions between the DTM mutants and SlREV in vitro and in vivo, DTM_L8F and DTM_L8F(L22A) had a weaker inhibition effect on SlREV-DNA binding than did wild type DTM.

**Fig. 6:**
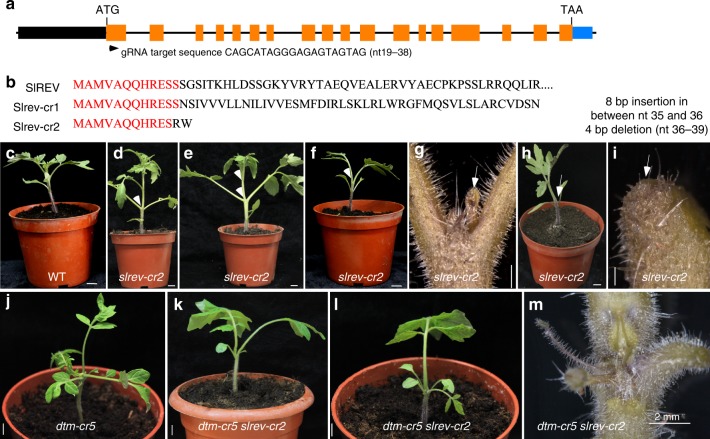
*SlREV* regulates shoot apical meristem (SAM) maintenance in tomato. **a** design of CRISPR-Cas9 editing for the tomato *REV* gene. The start codon ATG and the gRNA target sequence are indicated. The arrowhead indicates the gRNA target site. **b** deduced protein sequences of two *slrev* alleles created by CRISPR-Cas9. **c**–**i** representative images showing meristem defects of different severity observed in *slrev* mutants. *slrev-cr* mutants displayed weak (**d**) or strong (**e**) defects in leaf phyllotaxis (indicated by arrowheads). *slrev* seedlings often produced only one or two true leaves (**f**, **h**) with few small leaf-like tissues (**g**) or barren SAMs (**i**) (indicated by arrows). Since no allele-specific phenotypic abnormality was observed between *slrev-cr1* and *slrev-cr2*, only those of *slrev-cr2* are shown here. Scale bars represent 1 cm (**c**–**f**, **h**) and 100 μm (**g**, **i**), respectively. **j**–**l** seedling phenotypes of the *dtm slrev* double mutant. Scale bars, 1 cm. **m** dissected *dtm-cr5 slrev-cr2* apices showing SAM defects as revealed by stereomicroscopy. Scale bar, 2 mm

Since *dtm-1* seedlings produce ectopic axillary meristems, as observed in plants overexpressing *SlREV*^32^, we generated putative loss-of-function mutations in the *SlREV* gene using CRISPR-Cas9 (Fig. 6a, b). Two mutant alleles, *slrev-cr1* and *slrev-cr2*, exhibited SAM arrest and leaf phyllotaxis defects of differing severities, ranging from relatively normal SAM development, with an opposite leaf arrangement or two leaves (sometimes fused) with slowly initiated leaf primordia, to single leaves without shoot meristems (Fig. 6c–i). These developmental defects are indicative of premature stem cell consumption in the tomato *rev* mutants, contrasting with the overactive SAM activity in the *dtm* mutants. To further verify that DTM regulates SAM development through SlREV, we analyzed their genetic interaction by crossing *dtm-cr5* with *slrev-cr2* (both in the Moneymaker background). The resulting *dtm-cr5 slrev-cr2* seedlings were tricots, but looked more similar to *slrev-cr2* seedlings (Fig. 6j–m). This suggests that *DTM* regulates post-embryogenic SAM development in a *SlREV*-dependent manner, whereas its roles in embryogenesis is *SlREV*-independent.

Although the closest homolog of DTM, DTL, also interacts with tomato HD-ZIP III proteins, it has a different binding affinity, as revealed by yeast two hybrid and in vitro pulldown assays (Supplementary Fig. 9). Two *dtl* knockout mutants, created by CRISPR-Cas9 editing, had no obvious phenotypes (Supplementary Fig. 10a–g). Consistent with the phenotypic observation, *in situ* hybridization analysis suggested that *DTL* is likely not expressed in the SAM (Supplementary Fig. 10h–k). To test the possibility that *DTL* affects SAM development by interacting with *DTM*, we analyzed the phenotype of the double *dtm-cr5 dtl-cr1* mutant, and observed that it was indistinguishable from *dtm-cr5* (Supplementary Fig. 10l–n). These results suggest that *DTL* is not essential for post-embryogenic meristem development in tomato.

### *DTM-SlREV* defines expression domains of meristematic genes

**Fig. 7:**
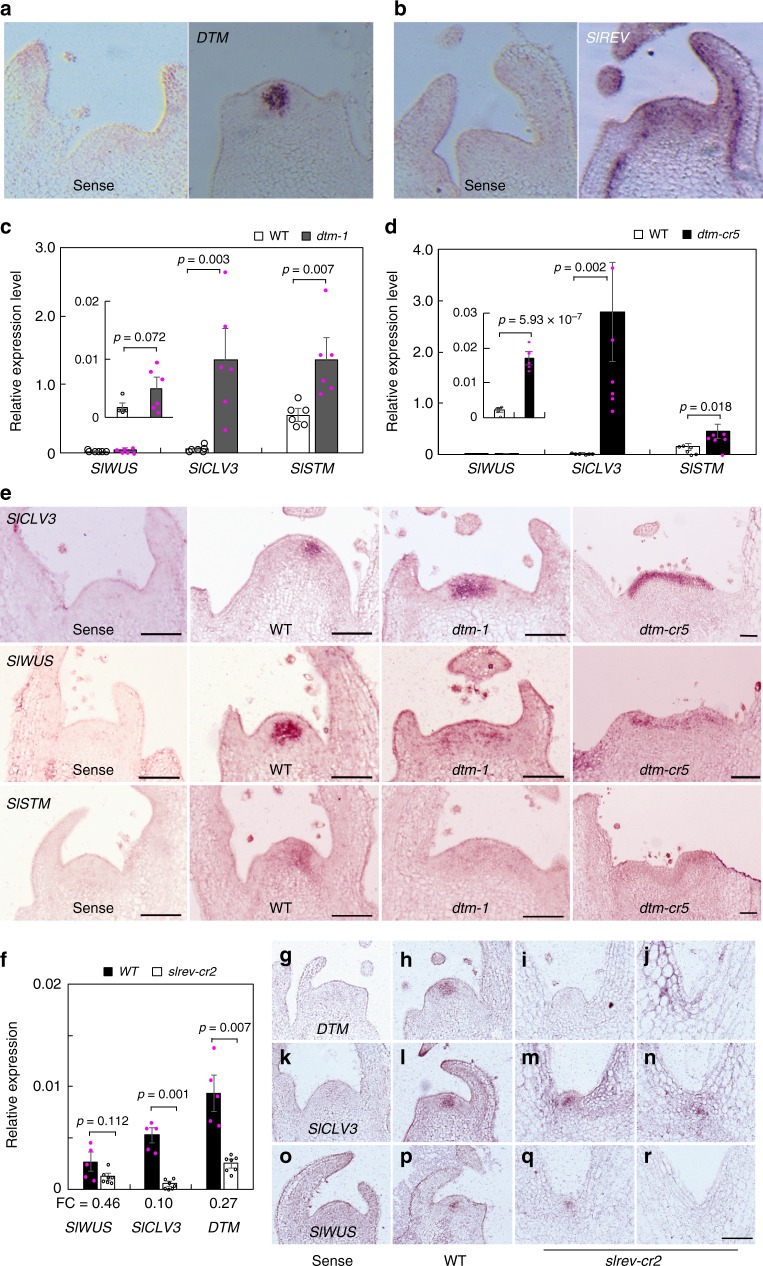
DTM defines the *SlCLV3* and *SlWUS* expression domains. **a**, **b** expression of *DTM* and *SlREV* in wild type (Moneymaker) shoot apices as revealed by RNA in situ hybridization. **c**, **d** expression patterns of the meristematic *SlCLV3*, *SlWUS* and *SlSTM* genes in the shoot apices of *dtm-1* (**c**), *dtm-cr5* (**d**), and their corresponding wild types (LA2397 and Moneymaker) at 6 days after germination (DAG) determined by quantitative reverse transcription (qRT)-PCR. *SleIF4α6* was used as the reference gene to normalize gene expression. Data represent means ± sem, *n* = 6 biologically independent samples. A two-tailed *t*-test was applied to compare the differences in means between *dtm-1* and wild type. **e** expression patterns of the *SlCLV3*, *SlWUS,* and *SlSTM* genes in the shoot apices of *dtm-1*, *dtm-cr5* and wild type (Moneymaker) at 6 DAG revealed by RNA *in situ* hybridization. Scale bars, 100 μm. **f** expression levels of *DTM* and the meristematic *SlCLV3*, *SlWUS,* and *SlSTM* genes in *slrev-cr2* shoot apices and wild type (Moneymaker) at 6 DAG determined by qRT-PCR. Data represent means ± sem, *n* = 5 (wild type) or 7 (*slrev-cr2*) biologically independent samples. Fold changes (FC, mutant vs wild type (WT)) of gene expression levels are given under the graph. A Welch’s *t*-test was applied to compare the differences in means between *slrev-cr2* and wild type. **g**–**r** expression of *DTM* (**g**–**j**), *SlCLV3* (**k**–**n**), and *SlWUS* (**o**–**r**) in *slrev-cr2* (**i**, **j**, **m**, **n**, **q** and **r**) and wild type (Moneymaker, **h**, **l** and **p**) shoot apices revealed by in situ hybridization. Hybridization using sense probes served as negative controls (**g**, **k** and **o**). Scale bar, 100 μm. The experiments were repeated at least twice using different batches of plants with similar results. Expression data collected from the *dtm-1* and *dtm-cr5* seedlings at 3 and 9 DAG are provided in Supplementary Fig. 10c-d. *SlSTM* expression in *slrev-cr2* and wild type apices is available in Supplementary Fig. 10e

Since *DTM* expression was detected mainly in the central zone of the SAM that marks the expression domains of *SlWUS* and *SlCLV3* (Fig. 7a)^29^, the observed SAM defects in the *dtm* mutants were likely caused by altered expression of these meristematic genes. We then monitored the expression patterns of the three meristem marker genes *SlCLV3*, *SlWUS,* and *SlSTM* in the shoot apices of *dtm-1*, *dtm-cr5,* and their corresponding wild types, at 3, 6 and 9 DAG. Expression analysis by quantitative reverse transcription PCR (qRT-PCR) revealed that *SlCLV3* transcript levels were substantially elevated in both the *dtm-1* and *dtm-cr5* shoot apices at all three time points, except for a marginal difference in expression level, which was detected between *dtm-cr5* and its wild type at 3 DAG (Fig. 7c–d; Supplementary Fig. 11a–d). *SlWUS* expression was also much higher in *dtm-cr5* shoot apices at all three time points and in *dtm-1* at 6 DAG, while *SlSTM* expression was apparently only upregulated in the two *dtm* alleles at 6 DAG.

Similarly, in situ hybridization analysis revealed that *SlCLV3* expression was higher and spatially expanded in the *dtm-1* and *dtm-cr5* shoot apices at 3, 6, and 9 DAG (Fig. 7e; Supplementary Fig. 12). In wild type, *SlCLV3* was expressed in the central zone of the SAM, apart from in the L1 layer. *SlCLV3* expression in the weak *dtm-1* allele was expanded but still detected in the central zone, whereas in the *dtm-cr5* null allele it was only detected in the outermost two layers of the whole apex, indicating that *DTM* restricts *SlCLV3* expression in the central zone. *SlWUS*, which is mainly expressed in the organizer zone, is likely also required for defining the size of the stem cell population in tomato^29,34^. In the *dtm-1* and *dtm-cr5* apices, *SlWUS* expression appeared patchy and expanded. Similarly, *SlSTM* expression was also affected. Unlike in wild type, the *SlSTM* expression in *dtm-1* and *dtm-cr5* apices was diffuse and no apparent expression domain could be defined.

Since DTM acts on SAM development in a SlREV-dependent manner, and the two genes have overlapping expression domains in the SAM (Fig. 7a, b), we examined the expression of *SlCLV3* and *SlWUS* in the SAMs of *slrev* seedling apices at 6 DAG. *SlCLV3* expression, measured by qRT-PCR, was substantially downregulated in *slrev-cr2*, and while there were indications that the expression of *SlWUS* was also slightly lower (Fig. 7f). We saw no evidence that *SlSTM* expression was affected by the *slrev* mutation (Supplementary Fig. 11e). Further, *in situ* hybridization analyses revealed that *SlCLV3* was expressed in a much deeper region of the SAM, likely in the organizer zone (Fig. 7k–n). The *SlWUS* expression level correlated with the severity of SAM defects; expression was not detected in the shoot apices without SAMs, but was maintained in the organizer zone with SAM-like structures (Fig. 7o–r). Consistent with previous results identifying *ZPR* genes as REV targets in *A. thaliana*^35^, *DTM* expression was downregulated in *slrev-cr2* shoot apices at 6 DAG (Fig. 7f–j). These results suggest that DTM and SlREV form a feedback loop to confine the expression of meristematic genes to specific cell layers in the SAM.

### Mutations in *DTM* impair leaf phyllotaxis

In addition to the defects in stem and axillary bud development, the leaf formation rate was also affected by *dtm* mutations, since five or more leaves were observed in *dtm-cr5* seedlings when the wild type had only three visible leaves (Fig. 3e, f). This likely reflects faster leaf initiation and the less serrated leaves in the *dtm-cr5* seedlings appeared to be clustered on a short stem region (Supplementary Fig. 13a, b). After removal of visible leaves, four small leaves or primordia forming on the *dtm-cr5* apices were observed by stereomicroscopy and SEM (Supplementary Fig. 12c, d). Although the leaf formation rate was less affected in *dtm-1*, longitudinal sectioning revealed that two precocious leaves formed almost simultaneously on the flank of the SAMs at 6 DAG (Supplementary Fig. 13e–g).

*LEAFLESS* (*LFS*) is required for leaf formation and its expression predicts leaf initiation^36^. Since *dtm* mutations affected leaf phyllotaxis, we used in situ hybridization to investigate whether *LFS* expression was altered in *dtm* mutants during leaf formation. Consistent with previous reports^36^, we observed that *LFS* was expressed in the incipient regions of leaf primordia. Although *LFS* expression remained unchanged in both the *dtm-1* and *dtm-cr5* apices at 3 DAG, its expression was barely detectable after this time point (Supplementary Fig. 13h-p). This effect on *LFS* expression is consistent with the observation that leaf formation was accelerated in *dtm-1* and *dtm-cr5* after germination.

## Discussion

It is well established that maintenance of SAM activity is primarily governed by the conserved CLV-WUS pathway^2^, but it is not well understood how this pathway is modified to allow diversified growth patterns in different plant species. In this study, we characterized a tomato LITTLE ZIPPER protein, DTM, and elucidated its role in SAM maintenance by defining *WUS* and *CLV3* expression domains.

Our data indicate that *DTM* is crucial for maintaining SAM activity in tomato, a model plant with a sympodial growth habit. Loss of, or weakened, DTM activity affects primary and secondary shoot meristem development, resulting in enlarged flattened SAMs, ectopic formation of axillary buds and leaves, and termination of SYMs on side shoots, as well as enlarged fasciated flowers and fruits. Such flower and fruit fasciation are often associated with failures in SAM maintenance, as observed in the tomato *fasciated inflorescence*, *fasciated and branched* and *fas* mutants^29^. Furthermore, the defects in SAM development caused by *dtm* mutations are characteristic features of *A. thaliana* mutants containing mutations in *CLV3* or genes involved in the transmission of the CLV3 peptide signal, such as *clavata1* and the quadruple mutant of the *CLAVATA3 INSENSITIVE RECEPTOR KINASE* genes^37–40^. Given that *dtm* mutants produce tricots, or fused cotyledons, *DTM* is also involved in embryogenic meristem formation. However, its primary role is to maintain post-embryogenic meristem development. The presence of multiple slightly domed SAM-like structures in the *dtm* seedlings indicates that *DTM* limits stem cell activity. Thus, impairing DTM activity causes overproduction of stem cells in the SAM. Under field growth conditions, the excessive number of stem cells ensures the development of extremely long inflorescences and large flowers.

Like tomato *dtm* mutants, the *A. thaliana* double mutant, *zpr3–2 zpr4–2*, shows a similar phenotypic abnormality resulting from SAM defects, including a high frequency of tricots, early leaf initiation and flower fasciation. This suggests that *DTM* and its *A. thaliana* homologs have conserved roles in SAM maintenance. However, DTM has an additional role in the regulation of cell differentiation in tomato because *dtm* mutations cause ectopic trichomes to be formed on the SAM epidermis, as also observed in the petunia (*Petunia hybrida*) and pepper (*Capsicum annuum*) *hairy meristem* mutants that have mutations in GRAS family genes, which encode proteins that interact with WUS homologs^41,42^. The regulation of cell differentiation by *DTM* is likely *CLV3*-independent, since ectopic trichomes are still formed on the SAM epidermis of the double *dtm-1 fas* mutants.

Consistent with a function in the regulation of SAM activity, *DTM* is mainly expressed in the central zone of the SAM that marks the *SlCLV3* and *SlWUS* expression domains^28,29^. Based on phenotypic observations and expression changes of these meristematic genes caused by the *dtm* mutations, we conclude that *DTM* regulates SAM maintenance through defining the expression domains of the meristem genes *SlCLV3* and *SlWUS*. Disrupting DTM activity leads to *SlCLV3* expression spreading to the L1 layer in the SAM, where it is not expressed in wild type^29^. Furthermore, high SlCLV3 activity likely causes the patchy *SlWUS* expression observed in *dtm* SAMs, a phenomenon that has been observed in *CLV3* overexpressing *A. thaliana*^9^.

Although previous studies have shown that *CLV3* restricts *WUS* expression to a limited set of cells in the central zone of the SAM in *A. thaliana*^9^, high *SlCLV3* expression caused by *dtm* mutations does not repress overall *SlWUS* transcription, consistent with a failure in CLV3 peptide signal transmission in the *dtm* mutants. Thus, the expanded *SlCLV3* expression caused by *dtm* mutations did not reduce SAM size, but instead led to enlarged SAMs. Such SAM enlargement, coupled with high *CLV3* expression, is well recognized in *A. thaliana* mutants such as *clavata1*, *receptor-like protein kinase2 2* and the quadruple mutant of the *CLAVATA3 INSENSITIVE RECEPTOR KINASE* genes, in which mutations disrupt the transmission of the CLV3 peptide signal^37–40^. Expanded *CLV3* and *WUS* expression has also been observed in the enlarged shoot meristems of an *A. thaliana* HD-ZIP III triple mutant^22^.

Our data also demonstrated that DTM can interact with HD-ZIP III transcription factors *in vitro* and in vivo, in agreement with previous studies in *A. thaliana*^25,27^. Both *dtm* and *zpr3–2 zpr4–2* have SAM defects similar to the *A. thaliana phv-1d* mutant^25,27^. Thus, DTM regulates SAM activity in tomato through a similar mechanism to *A. thaliana* ZPR proteins, by acting as a competitor to suppress HD-ZIP III transcription factor activity. Ectopic AM formation in *dtm* mutants is also observed in tomato plants overexpressing *miR166*-resistant *SlREV*^32^. Furthermore, loss-of-function mutations in the tomato *SlREV* gene result in opposite meristem phenotypes, including SAM arrest and disordered leaf phyllotaxis, suggesting that DTM controls SAM development mainly through suppression of SlREV activity. This conclusion is further supported by attenuated and domain-shifted expression of the meristematic genes *SlCLV3* and *SlWUS* in the SAMs of *slrev* seedlings. Moreover, the characteristic SAM defect phenotypes of the *dtm-cr5 slrev-cr2* mutants suggest that *DTM* acts in a *SlREV*-dependent manner. We further observed that DTM competes for HD-ZIP III binding in a dosage-dependent manner, supporting the hypothesis that ZPR proteins form heterotetramers, rather than heterodimers, with HD-ZIP III proteins^43^.

Given that HD-ZIP III transcription factors directly activate *WUS* and *STM* expression during de novo shoot regeneration and AM formation in *A. thaliana*^20,21^, we propose that the elevated expression of *SlWUS* and *SlSTM* in *dtm* apices is due to weak, or lost, inhibition of SlREV and other HD-ZIP III transcription factors that have yet to be identified. We conclude that a DTM-SlREV feedback loop plays an essential role in regulating the CLV-WUS signaling pathway by defining its expression domains.

## Methods

### Plant growth conditions and phenotypic analysis

*dtm-1* was identified by screening for defective meristem mutants in an ethyl methanesulfonate mutagenesis population in the cultivated tomato LA2397 background. Since *dtm-1* is female sterile, the mutant is maintained in a heterozygous state by backcrossing to wild type LA2397. Unless specified, all phenotypic observations and gene expression analysis were conducted with phytotron-grown seedlings or plants derived from selfed BC_2_ or BC_3_ heterozygotes. The phytotron growth conditions were maintained at 20–25 °C, a relative humidity of 70–80%, and 16 h daily illumination by 150 μmol m^−2^ s^−1^ light from metal halide and high-pressure sodium lamps. F_2_ mapping populations were grown under natural radiation in plastic greenhouses located in Songjiang, Shanghai.

### Genetic mapping of the *DTM* gene and sequence analysis

*dtm-1* was crossed with the wild relative *S. pimpinellifolium* accession LA1781 to produce a F_2_ mapping population. Based on the flower phenotypes of the mutant, 892 *dtm-1* plants identified from a total of 3,964 F_2_ progenies were used to map the *DTM* locus. Genomic DNA was extracted from 1–2 cm young leaves using a high throughput miniprep method^44^. Leaves were ground in 350 μL DNA extraction buffer [100 mM Tris, 5 mM EDTA, 150 mM sorbitol, 30 mM sodium bisulfite, 0.1% cetyltrimethylammonium ammonium bromide, 2% sarkosyl, pH 8.0] on a mixer mill MM 400 (Retsch). After extraction with 350 μL chloroform:isoamyl alcohol (24:1), DNA was precipitated with an equal volume of isopropanol from cleared supernatant by centrifugation and dissolved in 100 μL TE (10 mM Tris-Cl, 1 mM EDTA, pH 8.0). Molecular markers, comprising PCR and cleaved amplified polymorphic sequence markers, were developed based on single nucleotide polymorphisms and insertions/deletions identified comparison of genome sequence of cv. Heinz 1706 and *S. pimpinellifolium* LA1589, which were downloaded from SGN (https://solgenomics.net/). Marker information is provided in Supplementary Table 1.

After fine mapping, gene prediction was conducted in the 25.3 kb interval of the Heinz 1706 reference genome containing the *DTM* locus, using the FGENESH program in Softberry (http://www.softberry.com/). To identify the causal *dtm-1* mutation, genomic and complementary DNA (cDNA) sequences of the *DTM* genes from the mutant and the LA2397 wild type were determined by Sanger sequencing. Sequence assembly and comparisons were performed using the Sequencher® (Gene Codes Inc.) DNA analysis software.

To identify the tomato LITTLE ZIPPER gene family members, protein sequences of DTM and *A. thaliana* ZPR proteins were used as queries in a BLAST search of the ITAG2.5 genome database downloaded from SGN. Similarly, tomato HD-ZIP III proteins were identified using protein sequences of the five *A. thaliana* HD-ZIP III proteins as queries. Maximum parsimony trees of tomato and *A. thaliana* DTM/ZPR and HD-ZIP III proteins were constructed using MEGA7 (ver. 7.0.26)^45^. Phylogenetic relationships were tested with 1000 bootstrap replications and the trees were inferred using the Subtree-Pruning-Regrafting (SPR) method embedded in the program.

### Mutagenesis by CRISPR-Cas9

To create null *dtm* and *dtl* alleles using CRISPR-Cas9 genome editing technology, two single guide RNAs were designed using the CRISPR-P (http://cbi.hzau.edu.cn/crispr/) online tool^46^. After annealing, oligos were subsequently cloned into the *psgR-Cas9-At* vector^47^. Sequence-validated *psgR-Cas9-At* fragments containing guide sequences of the target genes were digested with the *Eco*RI and *Hind*III (New England BioLabs) restriction enzymes, and then cloned into the *pCAMBIA1300* binary vector. The *CRISPR-Cas9* vector used to create the *slrev-cr* mutants was constructed similarly, using a single guide RNA. These constructs were transformed into cv. Moneymaker (LA2706) by *Agrobacterium*-mediated transformation^48,49^. Briefly, cotyledons harvested from 7–10 days old seedlings were co-cultured for two days on Murashige and Skoog medium containing 100 μM acetosyringone with *Agrobacterium* strain GV3101 (OD_600_ = 0.5–0.6) harboring individual *CRISPR-Cas9* constructs in the dark under standard tissue culture conditions. Then, the transformants were selected on Murashige and Skoog medium supplemented with 15 mg L^−1^ hygromycin B (H370, PhytoTechnology Laboratories®). Plants of the T_0_ generation were genotyped by PCR using Cas9-specific primers and the mutations created by Cas9 were identified by sequencing. Cas9-free T_1_ plants harboring gene-edited mutations were backcrossed to Moneymaker, and their selfed F_2_ plants were used for phenotypic observations and gene expression analysis. Oligo and primer information used for vector construction and genotyping described here and hereafter is available in Supplementary Table 2.

### Microscopy of SAM morphology

*dtm-1*, *dtm-cr5,* and their corresponding wild type SAMs were dissected from seedlings at different developmental stages by removing any visible leaves and examined with a stereomicroscope (M125/DFC420, Leica). For SEM and histological analysis, the dissected meristems were fixed in formalin-acetic acid-alcohol (10% formaldehyde, 5% acetic acid, and 50% alcohol by volume). The meristems were gradually dehydrated by incubation in an ethanol series (30–100%). Half of these meristem samples were used for SEM analysis and so further dried using a critical-point dryer (Tousimis, USA), before being sputter coated in gold particles. SEM images were collected using a high-resolution field emission SEM (Zeiss Merlin Compact, Zeiss or JSM-6360LV, JEOL). The other half were embedded in Paraplast (Sigma-Aldrich) for histological analysis and 8 μm sections made using a Leica microtome (Leica). The sections were briefly stained with 0.05% toluidine blue.

### Gene expression analysis

Total *dtm-1*, *dtm-cr5*, and wild type RNA was extracted from shoot apices with visible leaves removed at specific stages using Trizol® reagent (ThermoFisher scientific)^48^. Residual genomic DNA was removed with DNAase I (NEB). To validate DTM coding sequences, 5′ and 3′ rapid amplification of cDNA ends was carried out using the SMARTer® RACE Kit (Clontech) following the supplier’s instructions. For qRT-PCR, 1 μg RNA was used to synthesize cDNA in a 20 μL reaction volume with the First Strand cDNA Synthesis kit (NEB). Two microliter 10fold-diluted cDNA was subjected to qRT-PCR with a 0.2 μM primer concentration. The PCR reaction containing 1× AceQ qPCR SYBR Green Master Mix (Vazyme Biotech, Nanjing) and 0.4 μL Rox Reference Dye 2, was performed using a QuantStudio3 (Applied Biosystems, Thermo) with the following PCR conditions: 45 cycles of 10 s at 95 °C and 30 s at 60^o^C. The *SleIF4α6* gene was used as a reference to normalize the expression levels of target genes^50^.

For in situ hybridization, dissected shoot apices were fixed in formalin-acetic acid-alcohol overnight at 4 °C. The fixed tissues were then embedded in Paraplast (Sigma-Aldrich) and 8 μm sections made using a microtome (Leica). After dewaxing and rehydration, in situ hybridization was performed as previously described using digoxigenin-labeled gene-specific probes^48^. Probes were prepared by in vitro transcription in the presence of 0.1 mM DIG-UTP (Roche) using 0.5–1.0 μg PCR amplified gene coding regions as DNA templates with the T7 promoter sequence incorporated. Sections were probed at 50 °C overnight in hybridization buffer [50% deionized formamide, 300 mM Tris pH 8.0, 100 mM sodium phosphate pH 6.8, 300 mM NaCl, 5 mM EDTA, 5× Denhardt’s solution, 5% dextran sulphate, 1 mg L^−1^ tRNA] containing gene-specific riboprobes of about 100 bp fragmented by carbonate hydrolysis. After post hybridization washes, gene expression was detected using anti-digoxigenin-AP Fab fragments (1:3000, # 11093274910, Roche) with nitro-blue tetrazolium and 5-bromo-4-chloro-3′-indolyphosphate (NBT/BCIP, Roche) as substrates for color detection. The primers used for qRT-PCR analysis and for preparing probe templates are listed in Supplementary Table 2.

### Bimolecular fluorescence complementation assay

The coding regions of *DTM*, the *DTM_L8F* mutant and the six tomato HD-ZIP III proteins (SlREV, SlPHB, SlPHV, SlHB8, SlHB15A, and SlHB15B) were amplified by PCR. The *DTM* sequences were then cloned into *pJW771* (*Pro35S:nLUC*) and the HD-ZIP III sequences into *pJW772* (*Pro35S:cLUC*), allowing expression of fusion proteins containing the N- and C-terminal halves of firefly luciferase, respectively^33^. For each interaction to be tested, equal amounts of *Agrobacterium* cells harboring the N- and C-terminal fusion expression cassettes were mixed. The cell mixture at an OD_600_ of 0.8 in infiltration buffer (10 mM MES, 10 mM MgCl_2_, 150 mM acetosyringone, pH 5.6) was infiltrated into *Nicotiana benthamiana* leaves using 1 mL needleless syringes^51^. Three days after infiltration, 1 mM Luciferin (Promega, WI) was infiltrated into the tobacco leaves. After 10 min incubation in the dark, luminescence was detected using an automatic chemiluminescence image analysis system (Tanon Science & Technology Co., Ltd., Shanghai) with an exposure time of 3 min.

### Yeast two hybrid assay

The *DTL* and *DTM* coding regions and those of its mutant forms *DTM_L8F*, *dtm-cr3*, *dtm-cr5*, and *dtm-cr7* were amplified by PCR and cloned into the *pBD-GAL4 Cam* bait vector (Stratagene). Similarly, the coding sequences (full-length or the N-terminal) of the six tomato HD-ZIP III genes were cloned into the *pAD-GAL4–2.1* prey vector (Stratagene). The bait and prey plasmids were co-transformed into yeast strain AH109 (Clontech) using the LiCl-PEG method according to the manufacturer’s instructions. After transformants were selected on synthetic dropout nutrient medium (SD/-Leu-Trp) plates, protein interactions were tested by cell growth on SD/-Trp-Leu-His and SD/-Trp-Leu-His-Ade plates with or without 1 mM 3-amino-1,2,4-triazole added to the media.

### Pulldown assay

Pulldown assays were conducted with *E. coli* expressed fusion proteins containing either cmyc (DTM and its mutant forms, DTL) or human influenza virus hemagglutinin (HA) tags (the six tomato HD-ZIP III proteins) and the Novagen® pET28 vector (Merck Millipore). Protein expression in *E. coli* BL21 star (DE3, Invitrogen) cells grown in liquid Luria-Bertani broth at an OD_600_ of 0.5–0.6 was induced with 1 mM isopropylthio-β-galactoside for 2 h at 28 °C. The cell pellets were collected by centrifugation at 12,000 × *g* for 2 min at 4 °C and resuspended in BugBuster Master Mix (Novagen) (200 μL per 1 mL cell culture). For soluble cmyc-tagged wild type DTL, DTM and its mutant forms as well as HA-tagged N-terminal portions of the HD-ZIP IIIs, the lysate was cleared by centrifugation at 14,000 × *g* for 15 min at 4 °C and directly used for immunoprecipitation. For HA-tagged full-length HD-ZIP III proteins that predominantly accumulated in protein inclusion bodies, the protein pellets collected by centrifugation at 14,000 × *g* for 15 min at 4 °C were solubilized in buffer A containing 10 mM Tris-HCl, pH 7.8, 50 mM NaCl, 1 mM EDTA, 6 M urea and 1 mM phenylmethanesulfonyl fluoride^52^, and then cleared by centrifugation at 12,000 × *g* for 15 min at 4 °C.

Pulldown assays were performed essentially as described in immunoprecipitation technical guide and protocols (Thermo Scientific) with some modifications. Before pulldown, 20 μL anti-HA Agarose resin (Sigma-Aldrich) (for a single reaction) was washed three times with 500 μL lysis buffer (10 m M HEPES, pH 7.5, 1.5 mM MgCl_2_, 10 mM KCl and 1 mM phenylmethanesulfonyl fluoride, 0.5% Triton X-100) containing 300 mM NaCl and once with 500 μL lysis buffer. The resin was suspended in 500 μL lysis buffer after the last wash and incubated with an aliquot of 50 μL freshly prepared lysate containing HA-HD-ZIP III proteins for 4 h at 4 °C. Anti-HA Agarose resin conjugated with HD-ZIP III fusion proteins was collected by a short centrifugation at 5000 × *g* at 4 °C. The resin was then washed three times with 500 μL lysis buffer containing 300 mM NaCl at 4 °C, followed by three washes with 500 μL lysis buffer containing 0.5% Triton X-100. After washing, the resin was suspended in 500 μL lysis buffer and incubated with 50 μL cmyc-DTM or cmyc-DTL lysate overnight at 4 °C. The resin containing the protein complex was then pelleted and washed as described above. The immunoprecipitation protein complex was released from the resin with 50 μL of 0.1 M glycine (pH2.5) for 30 min at 4 °C.

For Western blot analysis, IP prepared proteins and aliquots of input controls (2% of the amount used for each IP reaction) were separated in 10% (cmyc-DTL, -DTM and its mutants) or 12% (HA-HD-ZIP III proteins) SDS-PAGE gels and transferred to Amersham Hybond P 0.45 PVDF membranes (GE Healthcare Life Science). The membranes were subsequently blotted with primary anti-HA (1:1,000, # 3724S, CST) or anti-cmyc (1: 2,000, # A00172–40, Genscript) antibodies and then with secondary anti-rabbit HRP antibodies (1:5,000, # Ab6721, Abcam) as previously described^53^. To detect the antibody signals, the membranes were incubated in the buffer provided with the Super Signal West Pico Chemiluminescent substrate kit (Pierce). All full-length blots and gels are shown in Supplementary Fig. 14–17.

### Electrophoretic mobility shift assay

Electrophoretic mobility shift assay was performed essentially according to the procedures^48^ we established previously with some modifications. Briefly, 2 μg purified *SlREV* (nucleotide 1–795) plasmid (pTNT™ vector, Promega) and PCR fragments of *DTM*, *DTM_L8F*, and *DTM_L8F(L22A)* coding sequences with the SP6 and T7 polymerase promoter sequences incorporated were used as transcription templates in the Wheat Germ Protein Expression System (Promega). Individual proteins were then synthesized in 10 μL reaction volumes using the TnT™ SP6 High-Yield Wheat Germ Protein Expression System (# L3261, Promega) following the supplier’s instructions. For the DNA binding assay, 1 μL aliquots of the synthesized SlREV (1–265aa) protein was first mixed with specified amounts (0, 1, 2,and 4 μL) of wild type DTM protein and its mutant forms DTM_L8F and DTM_L8F(L22A) in gel-shift binding buffer from the LightShift EMSA Optimization and Control Kit (Thermo). After 20 min incubation on ice, the Cy5-labeled HB-9 DNA duplexes (5’-CAGATCTGTAATGATTACGAGAAT-3′) recognized by REV^25^ were added and the reactions were incubated at room temperature for another 20 min. The mixtures were then subjected to electrophoresis in a 6% native PAGE gel.

### Reporting Summary

Further information on experimental design is available in the Nature Research Reporting Summary linked to this article.

## Supplementary information

Reporting Summary

Description of additional supplementary items

Supplementary information

Supplementary Data 1

Supplementary Data 2

Supplementary Data 3

## Data Availability

Tomato sequence data for ZPR, HD-ZIP III, and other genes used in this study can be found at the Sol Genomics Network (SGN, https://solgenomics.net/) under the following accession numbers: DTM (Solyc09g009620), DTL (Solyc11g007100), SlZPR1 (Solyc01g091490), SlZPR2A (Solyc08g007570), SlZPR2B (Solyc08g079690), SlREV (Solyc11g069470), SlPHB (Solyc02g024070), SlPHV (Solyc02g069830), SlHB8 (Solyc08g066500), SlHB15A (Solyc03g120910), SlHB15B (Solyc12g044410), SlWUS/LC (Solyc02g083950), SlCLV3/FAS (Solyc11g071380), SlSTM (Solyc02g081120), LFS (Solyc05g013540). *A. thaliana* ZPR and HD-ZIP III gene sequences can be found at the Arabidopsis Information Resource (TAIR, https://www.arabidopsis.org/index.jsp): ZPR1 (At2g45450), ZPR2 (At3g60890), ZPR3 (At3g52770), ZPR4 (At2g36307), ATHB15 (At1g52150), ATHB8 (At4g32880), REV (At5g60690), PHB (At2g34710) and PHV (At1g30490). Source data of floral organ number measurements used for making the boxplot in Fig. 1f is shown in Supplementary data 1, and source data for expression analysis by qRT-PCR in Fig. 7 and Supplementary Fig. 10 is available in Supplementary data 2 and 3, respectively. All other data generated in this study are available from the authors upon request.
