# Supplementary material for: Domain-specific expression of meristematic genes is defined by the LITTLE ZIPPER protein DTM in tomato
Source: Commun Biol. 2019 Apr 23;2:134. doi: 10.1038/s42003-019-0368-8 (PMC6478692; doi:10.1038/s42003-019-0368-8)
Supplement: Supplementary file 1 — Reporting Summary [file 42003_2019_368_MOESM1_ESM.pdf]

nature research

Corresponding author(s): Han Xiao

Last updated by author(s): Jan 31, 2019

Reporting Summary

Nature Research wishes to improve the reproducibility of the work that we publish. This form provides structure for consistency and transparency in reporting. For further information on Nature Research policies, see Authors & Referees and the Editorial Policy Checklist.

Statistics

For all statistical analyses, confirm that the following items are present in the figure legend, table legend, main text, or Methods section.

n/a	Confirmed	
☐	☒	The exact sample size (n) for each experimental group/condition, given as a discrete number and unit of measurement
☐	☒	A statement on whether measurements were taken from distinct samples or whether the same sample was measured repeatedly
☐	☒	The statistical test(s) used AND whether they are one- or two-sided<br>*Only common tests should be described solely by name; describe more complex techniques in the Methods section.*
☒	☐	A description of all covariates tested
☒	☐	A description of any assumptions or corrections, such as tests of normality and adjustment for multiple comparisons
☐	☒	A full description of the statistical parameters including central tendency (e.g. means) or other basic estimates (e.g. regression coefficient) AND variation (e.g. standard deviation) or associated estimates of uncertainty (e.g. confidence intervals)
☐	☒	For null hypothesis testing, the test statistic (e.g. F, t, r) with confidence intervals, effect sizes, degrees of freedom and P value noted<br>*Give P values as exact values whenever suitable.*
☒	☐	For Bayesian analysis, information on the choice of priors and Markov chain Monte Carlo settings
☒	☐	For hierarchical and complex designs, identification of the appropriate level for tests and full reporting of outcomes
☒	☐	Estimates of effect sizes (e.g. Cohen's d, Pearson's r), indicating how they were calculated

Our web collection on statistics for biologists contains articles on many of the points above.

Software and code

Policy information about availability of computer code

Data collection	No software was used for data collection.
Data analysis	Detailed information of softwares are described in method. MEGA7 (ver. 7.0.26) was used for phylogenetic analysis, and Sequencher 4.10.1 (Gene Codes Inc.) was used for sequence analysis. FGENESH program in Softberry (http://www.softberry.com/) was used for gene prediction.

For manuscripts utilizing custom algorithms or software that are central to the research but not yet described in published literature, software must be made available to editors/reviewers. We strongly encourage code deposition in a community repository (e.g. GitHub). See the Nature Research guidelines for submitting code & software for further information.

Data

Policy information about availability of data

All manuscripts must include a data availability statement. This statement should provide the following information, where applicable:

- Accession codes, unique identifiers, or web links for publicly available datasets
- A list of figures that have associated raw data
- A description of any restrictions on data availability

Tomato sequence data of ZPR, HD-ZIP III and other genes used in this study can be found from Sequence data associated this article can be found in the Arabidopsis Genome Initiative or Sol Genomics Network databases (SGN, https://solgenomics.net/) under the following accession numbers: DTM (Solyc09g009620), DTL (Solyc11g007100), SlZPR1 (Solyc01g091490), SlZPR2A (Solyc08g007570), SlZPR2B (Solyc08g079690), SlREV (Solyc11g069470), SlPHB (Solyc02g024070), SlPHV (Solyc02g069830), SlHB8 (Solyc08g066500), SlHB15A (Solyc03g120910), SlHB15B (Solyc12g044410), SlWUS/LC (Solyc02g083950), SlCLV3/FAS (Solyc11g071380), SlSTM (Solyc02g081120), LFS (Solyc05g013540). Arabidopsis ZPR and HD-ZIP III genes can be found from, the Arabidopsis Information Resource (TAIR, https://www.arabidopsis.org/index.jsp): ZPR1 (At2g45450), ZPR2 (At3g60890), ZPR3 (At3g52770), ZPR4 (At2g36307), ATHB15 (At1g52150), ATHB8 (At4g32880), REV (At5g60690), PHB (At2g34710) and PHV (At1g30490). Source data of floral organ number measurements used for making the boxplot in Fig. 1f is available in

Supplementary data 1, and source data for expression analysis by qRT-PCR in Fig. 7 and Supplementary Fig. 10 is available in Supplementary data 2 and 3, respectively. All other data generated in this study are available from the authors upon request.

Field-specific reporting

Please select the one below that is the best fit for your research. If you are not sure, read the appropriate sections before making your selection.

☒ Life sciences ☐ Behavioural & social sciences ☐ Ecological, evolutionary & environmental sciences

For a reference copy of the document with all sections, see nature.com/documents/nr-reporting-summary-flat.pdf

Life sciences study design

All studies must disclose on these points even when the disclosure is negative.

Sample size	No statistical methods were employed to predetermine sample size because dtm mutants showed distinctive phenotypes. For phenotypic analysis,10-15 plants per genotype were examined. At least 10 SAMs per genotype per time point were used for RNA In situ hybridization, histological and SEM experiments.
Data exclusions	No data were excluded.
Replication	qRT-PCR analysis of gene expression in dtm-1 mutants was conducted with at least three biological replicates and repeated at least two times with different batches of plants.
Randomization	Samples were collected randomly. Seeds harvested from heterozygous plants (dtm/+) were sowed without pre-genotyping, and genotypes were deterimed after germination. Then, sampled by genotypes.
Blinding	We performed our protein interaction experiments blindly by at least two people, two performed yeast two hybrid and one performed Pulldown assay. For other experiments, blinding was not feasible due to that the dtm mutants have distinctive phenotypes from wild type.

Reporting for specific materials, systems and methods

We require information from authors about some types of materials, experimental systems and methods used in many studies. Here, indicate whether each material, system or method listed is relevant to your study. If you are not sure if a list item applies to your research, read the appropriate section before selecting a response.

Materials & experimental systems

n/a	Involved in the study	
☐	☒	Antibodies
☒	☐	Eukaryotic cell lines
☒	☐	Palaeontology
☒	☐	Animals and other organisms
☒	☐	Human research participants
☒	☐	Clinical data

Methods

n/a	Involved in the study	
☒	☐	ChIP-seq
☒	☐	Flow cytometry
☒	☐	MRI-based neuroimaging

Antibodies

Antibodies used	Anti-HA, Cell signaling technology, #3724<br>Anti-cmyc, Genescript, #A00172-40<br>secondary anti-rabbit HRP antibodies, Abcam, Ab6721<br>anti-digoxigenin-AP Fab fragments, # 11093274910, Roche
Validation	Anti-HA, 1:1000<br>Anti-cmyc, 1:2000<br>anti-rabbit HRP antibodies; 1:5000<br>anti-digoxigenin-AP Fab fragments; 1:3000,<br>All antibodies were used in Western blots or in situ hybridizations including appropriate controls.
