# Supplementary material for: Domain-specific expression of meristematic genes is defined by the LITTLE ZIPPER protein DTM in tomato
Source: Commun Biol. 2019 Apr 23;2:134. doi: 10.1038/s42003-019-0368-8 (PMC6478692; doi:10.1038/s42003-019-0368-8)
Supplement: Supplementary file 2 — Description of additional supplementary items [file 42003_2019_368_MOESM2_ESM.docx]

**Description of additional supplementary files**

**File Name**: Supplementary Data 1

**Description**: Source data of floral organ number measurements of *dtm-1* and wild type used in Fig. 1f.

**File Name**: Supplementary Data 2

**Description**: Source data of gene expression analysis of *dtm-1*, *dtm-cr5* and wild types used in Fig. 7c,d,f.

**File Name**: Supplementary Data 3

**Description**: Source data of gene expression analysis of *dtm-1*, *dtm-cr5*, *slrev-cr2* and wild types used in Supplementary Fig. 10.
