# Supplementary material for: Domain-specific expression of meristematic genes is defined by the LITTLE ZIPPER protein DTM in tomato
Source: Commun Biol. 2019 Apr 23;2:134. doi: 10.1038/s42003-019-0368-8 (PMC6478692; doi:10.1038/s42003-019-0368-8)
Supplement: Supplementary file 3 — Supplementary information [file 42003_2019_368_MOESM3_ESM.pdf]

a

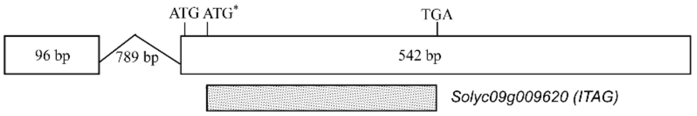

b

```
         1              16                                 50
FGENESH  MDRINSKLYLQNCYIMAENERLRKKAELLNQENQQLLSELKHRLSQAGPS
ITAG                    MAENERLRKKAELLNQENQQLLSELKHRLSQAGPS

FGENESH  NNNNNNNNNGGGKNNSIPDLNLTNCSSSKNASSKSKKK
ITAG     NNNNNNNNNGGGKNNSIPDLNLTNCSSSKNASSKSKKK
```

Supplementary Fig. 1 Correction of the ITAG annotation for *Solyc09g009620*. a, genomic structure of the *Solyc09g009620/DTM* gene. The ITAG-annotated *Solyco9g009620* (shaded) has single exon and the second ATG (indicated by *) is predicted as the start codon. But, analysis of 5’ RACE and 3’ RACE sequences revealed that *Solyc09009620* contains two exons, and the first ATG in the second exon has been predicted as start codon by gene prediction program FGENESH. **b**, alignment of protein sequences deduced from gene prediction by FGENESH and ITAG. The 15 amino acid residues missed in ITAG-annotation are highlighted in red.

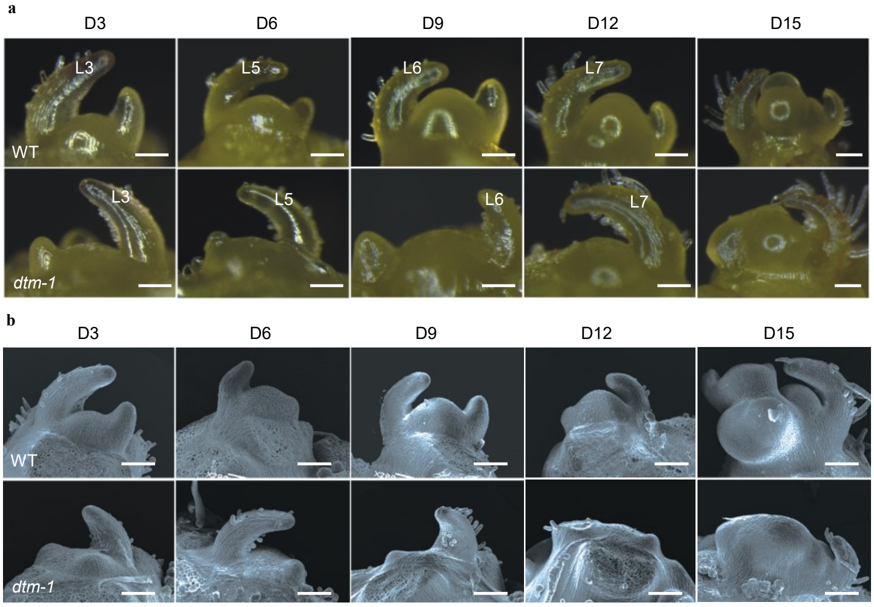

Supplementary Fig. 2 Doming of *dtm-1* SAMs and wild type. SAM doming was observed under a stereomicroscope (**a**) or by SEM (**b**). SAM morphology were examined every three days after germination (D3-D15 as shown above each column of micrographs), and leaves were numbered from old to young. Scale bars, 100 µm.

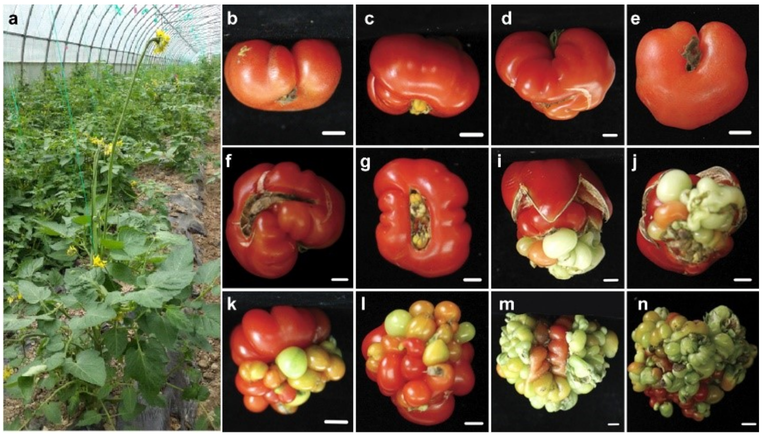

Supplementary Fig. 3 Variations in fruit morphology observed in *dtm-1* plants of a F_2 population derived from a cross between *S. pimpinellifolium* LA1781 and *dtm-1*. a, extremely elongated flower stalks found in *dtm-1* plants grown in plastic greenhouse. **b-n**, an array of *dtm-1* fruits with different severities of fasciation. Extremely elongated flower stalks (**a**) and fasciated fruits (**i-n**) were only observed in plants grown in plastic greenhouse. Scale bars, 1 cm.

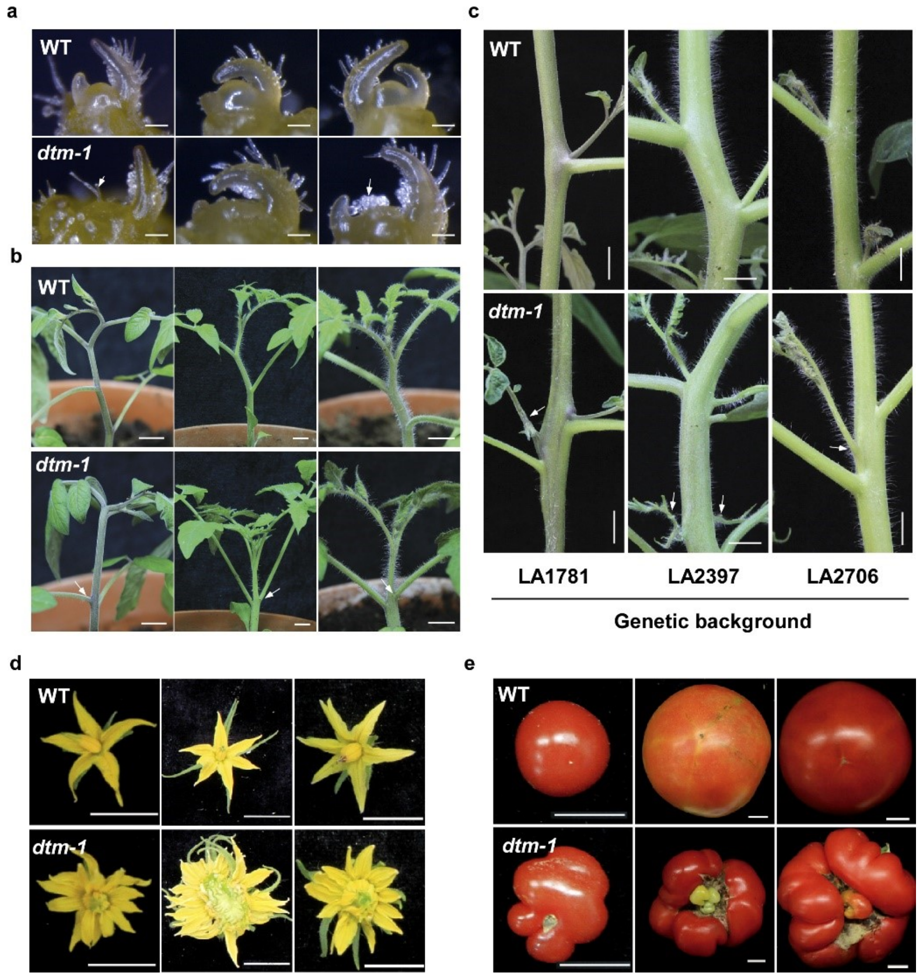

Supplementary Fig. 4 The *dtm-1* allele impacts SAM development independent on genetic background. **a**, images of dissected vegetative SAMs from *dtm-1* NILs in LA1781, LA2397 and LA2706 (Moneymaker) backgrounds. Ectopic trichomes formed on the dooms were indicated by arrows. Scale bar, 100 μm. **b**, photographs showing altered leaf phyllotaxis (indicated by arrows) of *dtm-1* plants in LA1781, LA2397 and LA2706 backgrounds. Scale bar, 1 cm. **c**, photographs of shoot segments showing mis-formed axillary buds from *dtm-1* plants in LA1781, LA2397 and LA2706 backgrounds. **d-e**, flower and fruit phenotypes of *dtm-1* allele in LA1781, LA2397 and LA2706 backgrounds. Scale bar, 1 cm. The *dtm-1* mutant in LA2397 background was crossed to LA1781 and LA2706, and backcrossed four times to wild type parents before they are subjected to phenotypic analysis. Images in (**a-e**) are arranged in the same way as indicated in (**c**).

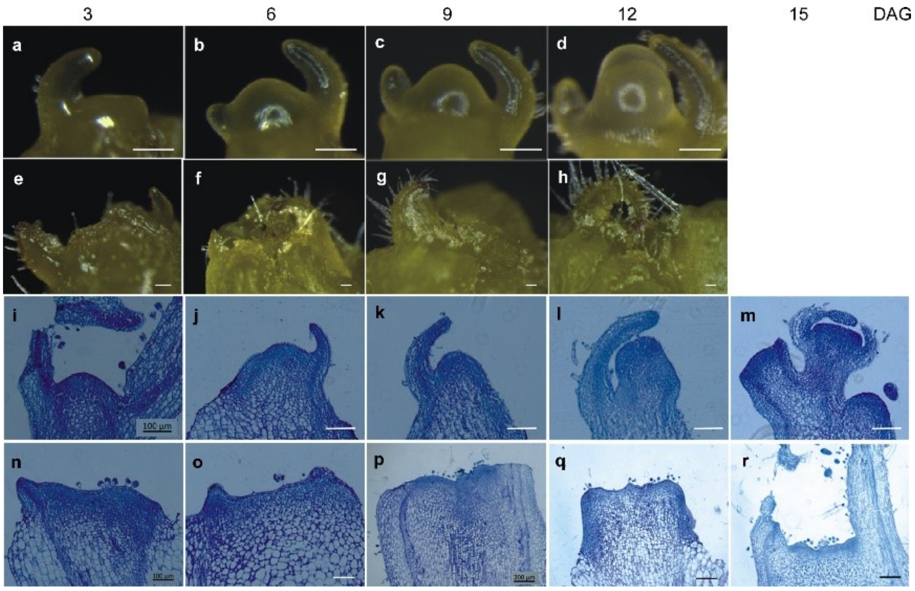

Supplementary Fig. 5 SAM morphology of *dtm-cr5* and wild type. a-h, stereomicroscopic images of *dtm-cr5* (**a-d**) and wild type (Moneymaker, **e-h**) SAMs at 3, 6, 9 and 12 days after germination (DAG). Scale bars, 100 μm. **i-r**, parafilm sections of *dtm-cr5* (**n-r**) and wild type (**i-m**) shoot apices at 3, 6, 9, 12 and 15 DAG. Scale bars represent 100 μm (**i-o**) and 200 μm (**o-r**), respectively.

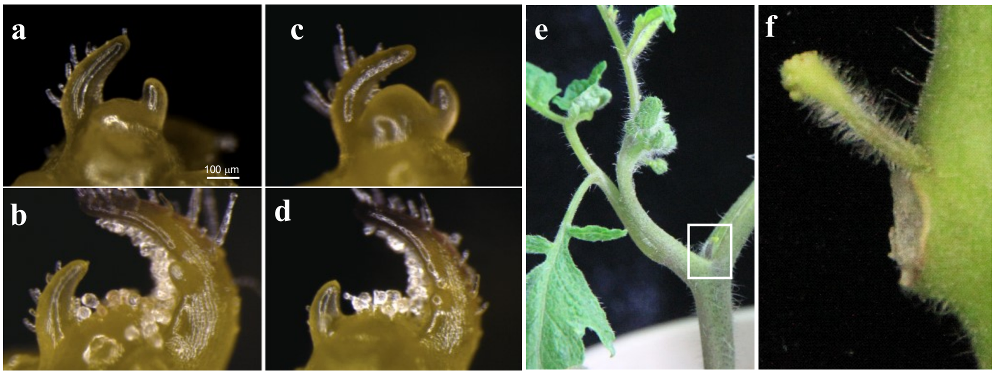

Supplementary Fig. 6 Genetic interaction between *FAS* and *DTM*. a-d, SAM morphology of *dtm-1* (**b**), *fas* (**c**), *dtm-1 fas* (**d**) and wild type (**a**) was examined at 6 DAG under a stereomicroscope. Scale bar, 100 µm. **e**, image of fas dtm-1 stem showing defect in axillary shoot development. **f**, close-up of the selected region indicated in (e) by the white rectangle. *dtm-1* in LA2397 background was crossed to cv. Super Beefsteak containing the *fas* mutation in the *SlCLV3* gene, and the four genotypes were identified in F_2 progenies from this cross.

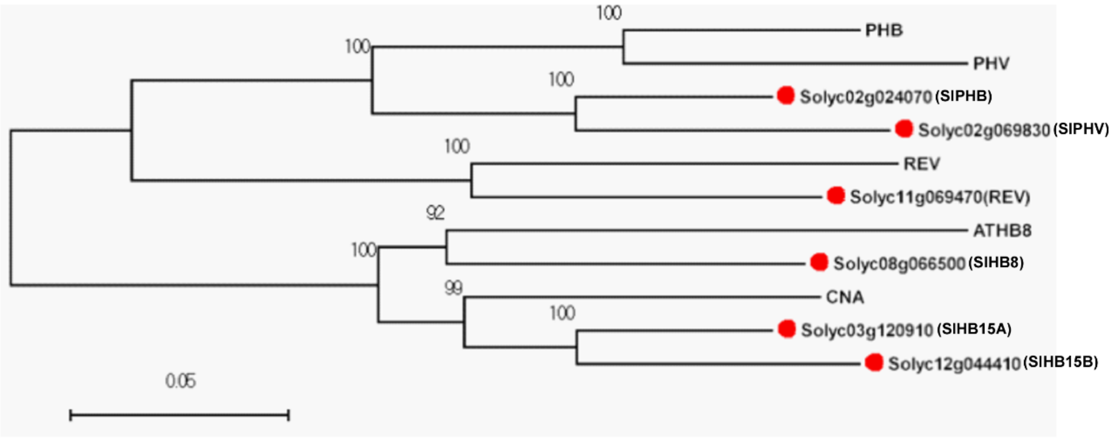

Supplementary Fig. 7 Phylogenetic analysis of HD-ZIP III proteins from tomato and Arabidopsis by MEGA7. The consensus phylogenetic tree was generated by 1,000 bootstrap replications. The numbers next to branches represent the percentages of the replicate trees (only 50% or higher reported) in the bootstrap test.

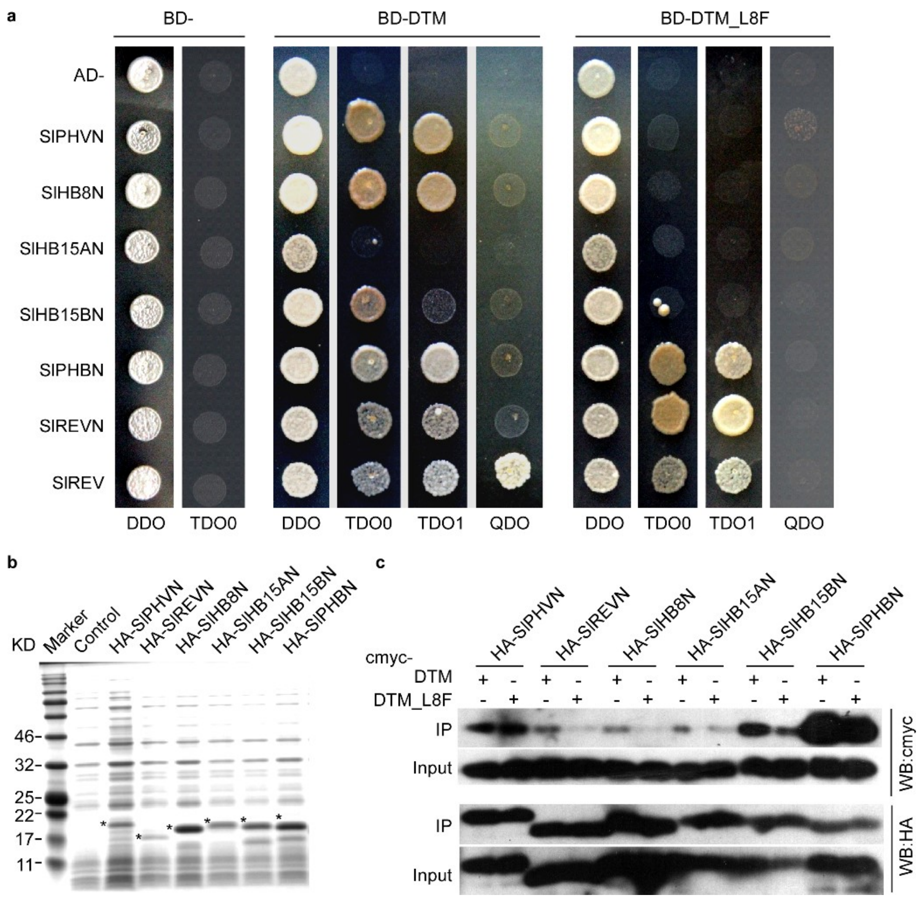

Supplementary Fig. 8 Interactions between DTM, DTM_L8F and the N-terminals of tomato HD-ZIP III proteins. a, DTM and DTM_L8F interacts with the N-terminals of the six tomato HD-ZIP III proteins in yeast. The full-length SlREV was included as control. **b**, expression of the N-terminal portions of individual tomato HD-ZIP IIIs in *E. coli*. The N-terminal portions of tomato HD-ZIP III proteins consist of the first 127 (SlPHV) or 132 aa (the remaining five) containing homeodomain and leucine zipper domain. *, indicates the fusion protein induced. **c**, comparison of binding specificity between DTM and DTM_L8F to the N-terminals of the six tomato HD-ZIP III proteins by pulldown assay. DTM and DTM_L8F fused to cmyc were tested for their binding affinities with the N-terminal portions of HD-ZIP III proteins tagged by HA. DDO, SD-Leu-Trp; TDO0 and TDO1, SD-Leu-Trp-His with 0 and 1 mM 3-amino-1,2,4- triazole (3-AT), respectively; QDO, SD-Leu-Trp-His-Ade. IP, immunoprecipitation; WB, Western blot.

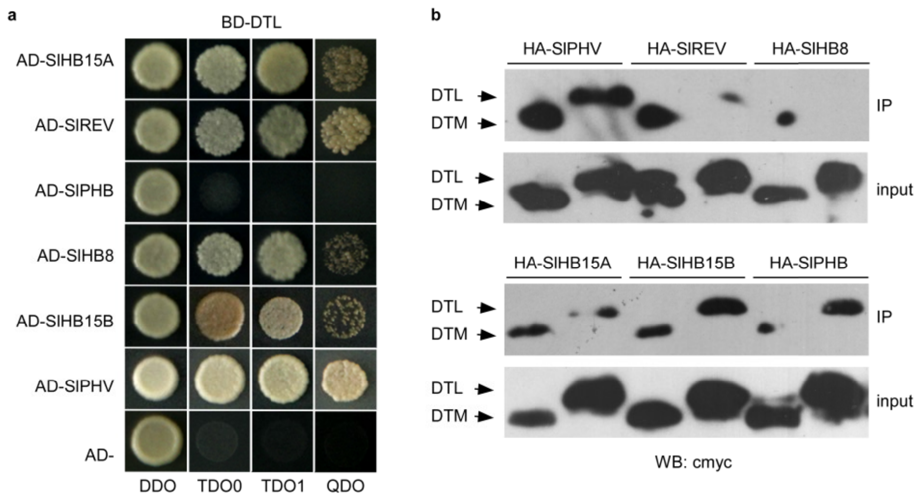

Supplementary Fig. 9 Interactions between DTL and tomato HD-ZIP III proteins. **a**, DTL interacts members of tomato HD-ZIP III proteins in yeast. **b**, comparison of binding specificity between DTM and DTL to tomato HD-ZIP III proteins by pulldown assay. DTM and DTL fused to cmyc were tested for their binding affinities with HD-ZIP III proteins tagged by HA. DDO, SD-Leu-Trp; TDO0 and TDO1, SD-Leu-Trp-His with 0 and 1 mM 3-amino-1,2,4- triazole (3-AT), respectively; QDO, SD-Leu-Trp-His-Ade. IP, immunoprecipitation; WB, Western blot.

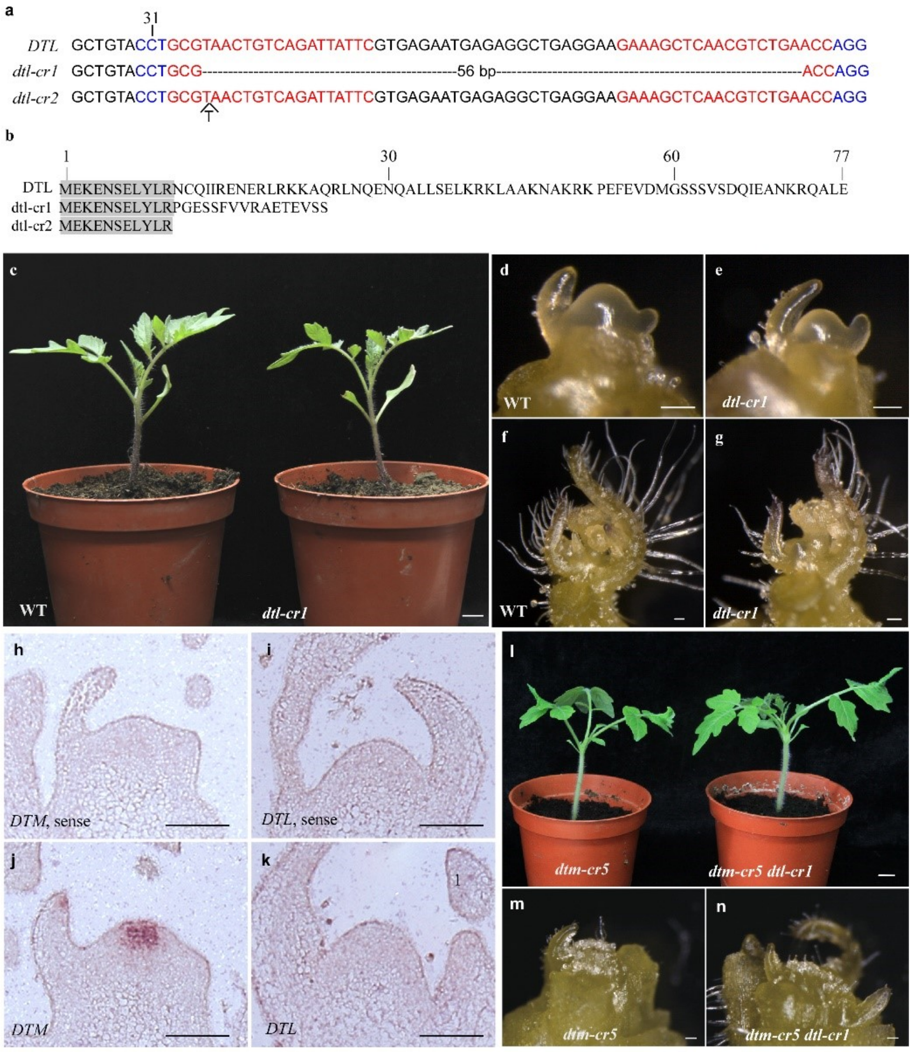

Supplementary Fig. 10 Loss-of-function in *DTL* has no visible SAM phenotype. a, two putative loss-of-function *dtl* alleles created by CRISPR-Cas9 were identified by Sanger sequencing. The two gRNA target sequences are highlighted in red. **b**, dedued amino acid sequences encoded by the two edited alleles *dtl-cr1* and *dtl-cr2*. Identical sequences between the mutants and wild type DTL are shaded in grey. **c**, images of *dtl-cr1* and wild type seedlings. **d-g**, SAM morphology of *dtl-cr1* (**e**, **g**) and wild type (**d**, **f**) at vegetative (**d**, **e**) and reproductive phases (**f**, **g**). **h-k**, DTM and DTL expression in SAMs at 6 days after germination (DAG). **l-n**, seedling (**h**) and SAM (**i**, **j**) phenotypes of *dtm-cr5* and *dtm-cr5 dtl-cr1*. The double mutant *dtm-cr5 dtl-cr1* displayed the same developmental defects with *dtm-cr5* in SAM development and leaf phyllotaxis. Scale bars represent 1 cm (**c**, **l**) and 100 μm (**d-k**, **m**, **n**), respectively.

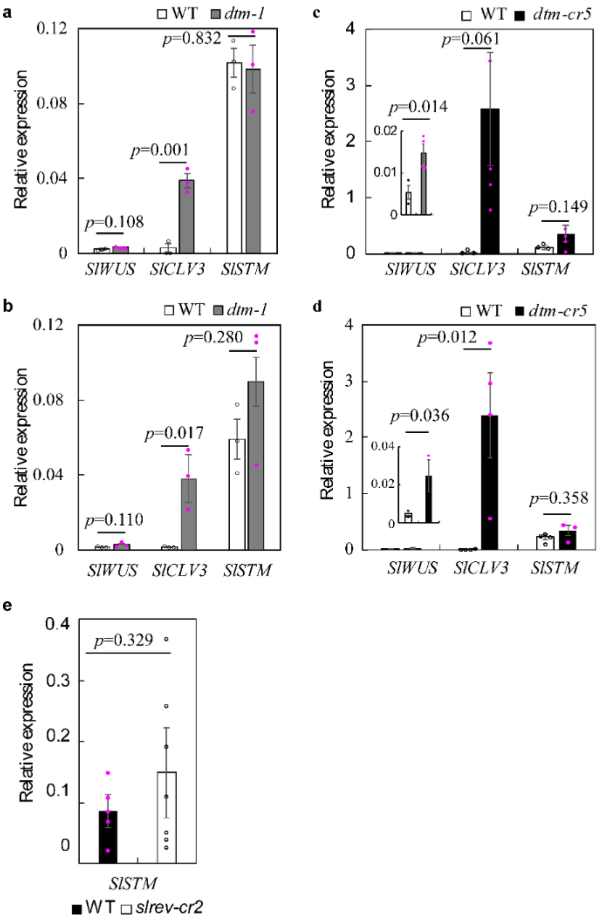

Supplementary Fig. 11 Expression levels of meristematic genes in the shoot apices of *dtm-1*, *dtm-cr5*, *slrev-cr2* and their wild types. a-d, relative expression levels of meristematic genes *SlCLV3*, *SlWUS* and *SlSTM* in the shoot apices of *dtm-1* (**a**, **b**) and *dtm-cr5* (**c**, **d**) seedlings at 3 (**a**, **c**) and 9 (**b**, **d**) DAG. **e**, *SlSTM* expression in *slrev-cr2* and wild type (Moneymaker) shoot apices at 6 DAG. The experiments were repeated at least twice using different batches of plants with similar results. Data are reported as means ± SE of three-seven biological replicates. A Welch's t-test (comparison between two groups with unequal sample sizes) or two tailed t-test was applied to compare the differences in means between mutant and wild type. LA2397 and Moneymaker (LA2706) were used as respective wild type control of *dtm-1* and *dtm-cr5*. Individual measurements of expression relative to that of reference gene *SleIF4α6* were indicated by dots or small cycles. Expression data collected from *dtm-1* and *dtm-cr5* apices at 6 DAG are presented in **Fig. 7c**, **d**.

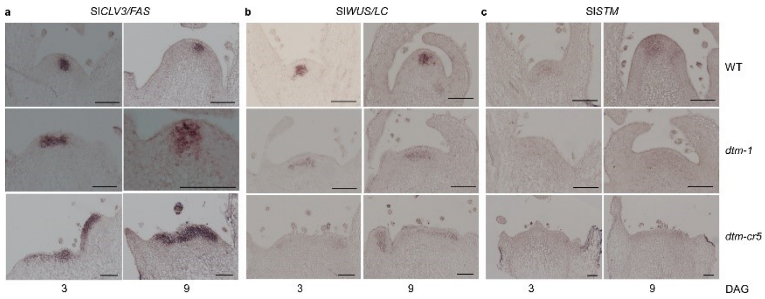

Supplementary Fig. 12 Expression patterns of meristematic genes in the shoot apices of *dtm* mutants and wild type revealed by *in situ* hybridization. Expression of *SlCLV3* (**a**), *SlWUS* (**b**) and *SlSTM* (**c**) in the shoot apices of *dtm-1*, *dtm-cr5* and wild type (Moneymaker) at 3 and 9 DAG. Scale bars, 100 μm. Data collected from the seedlings at 6 DAG are provided in **Fig. 7e**.

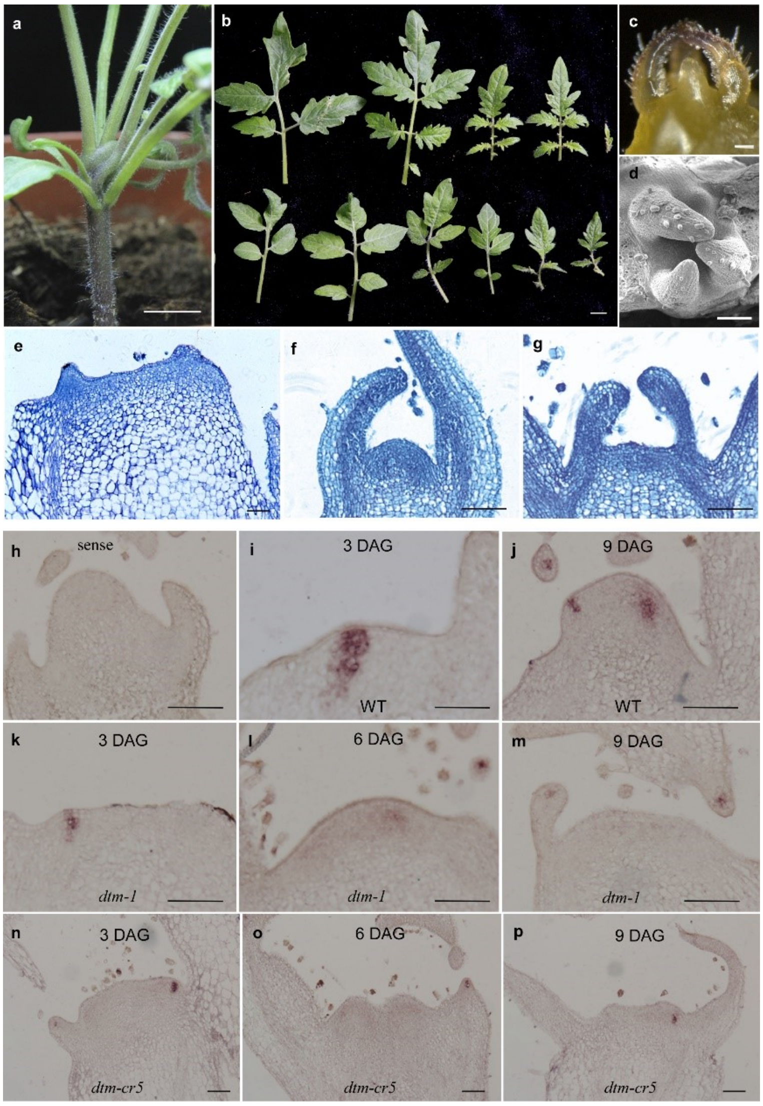

Supplementary Fig. 13 *DTM* is involved in regulation of leaf initiation. a, an image showing multiple leaves clustered at the top of the seedlings. Scale bar, 1 cm. **b**, leaf development in *dtm-cr5* and wild type. Leave were dissected from three-week-old plants. Scale bar, 1 cm. **c-e**, early leaf initiation in *dtm-cr5* seedlings by stereomicroscopy (**c**), SEM (**d**) and parafilm section (**e**). Scale bar, 100 μm. **f**, parafilm section of wild type apices at 6 DAG. **g**, parafilm section of *dtm-1* apices at 6 DAG. Scale bars, 100 μm. **h-p**, *LFS* expression by RNA *in situ* hybridization in the shoot apices of *dtm* mutants and wild type at 3, 6 and 9 DAG. Scale bars, 100 μm.

Blots used in Fig 4c

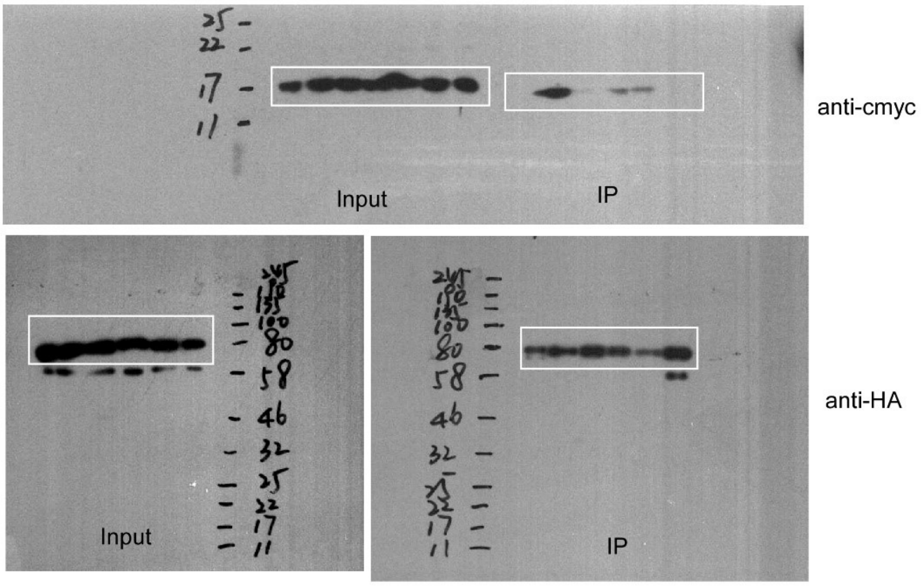

Blots used in Fig 4d

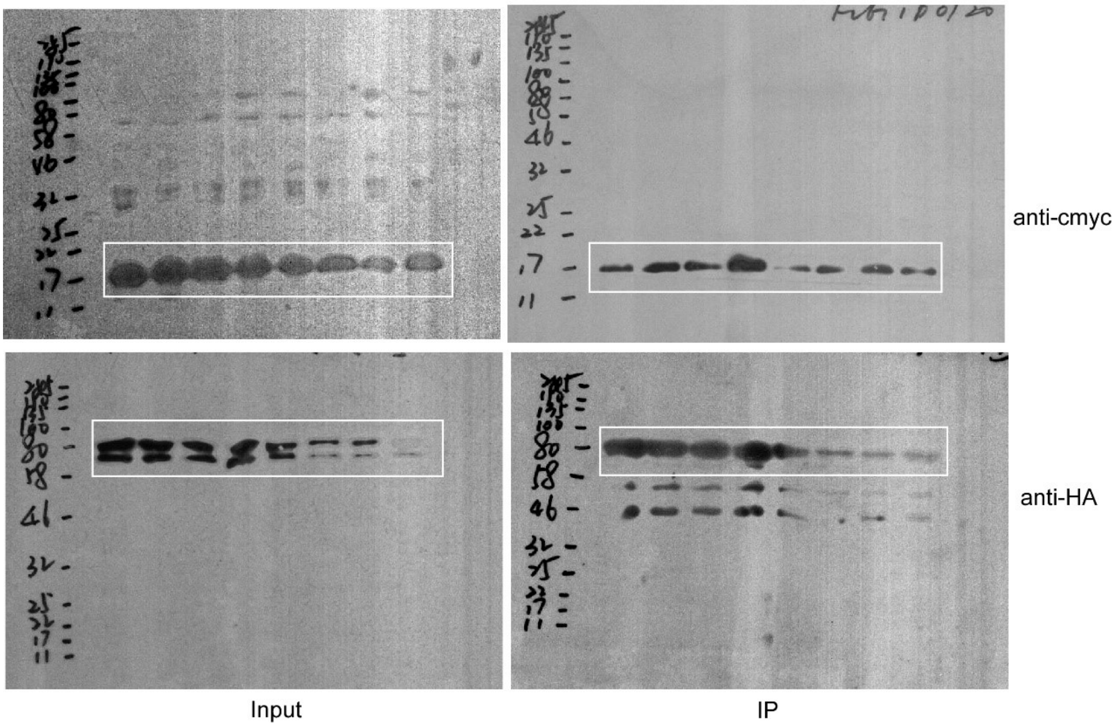

Supplementary Figure 14 Original blot images used in Fig. 4

The panels boxed were used in Fig. 4c,d. The size (kDa) of the marker proteins are marked on the left or right of each image.

Blots used in Fig 5c

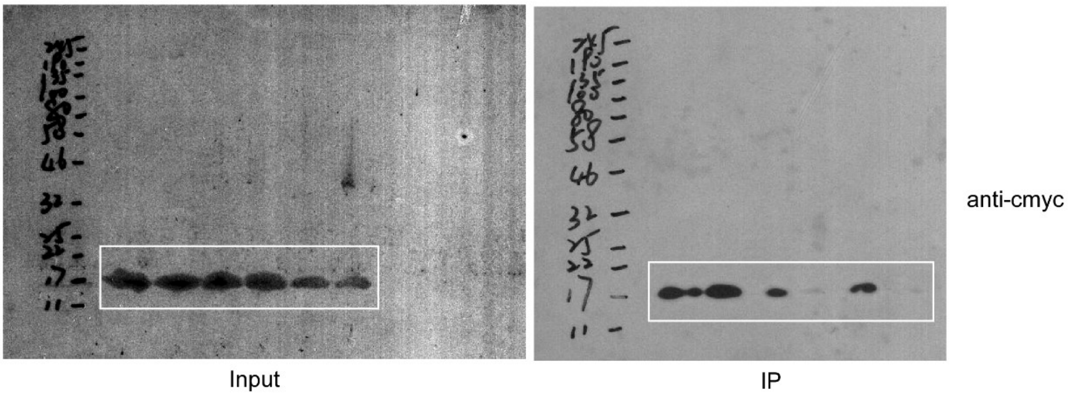

Blots used in Fig 5d

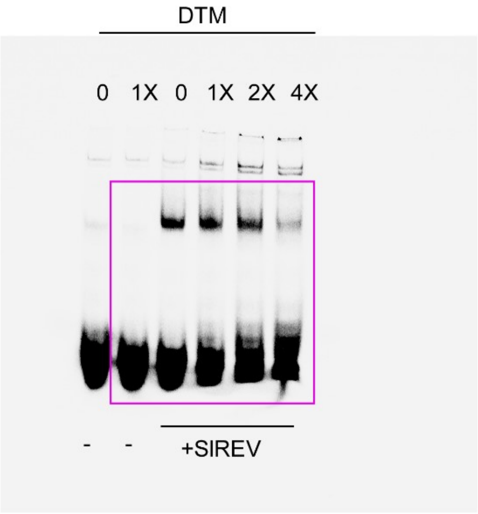

Blots used in Fig 5e

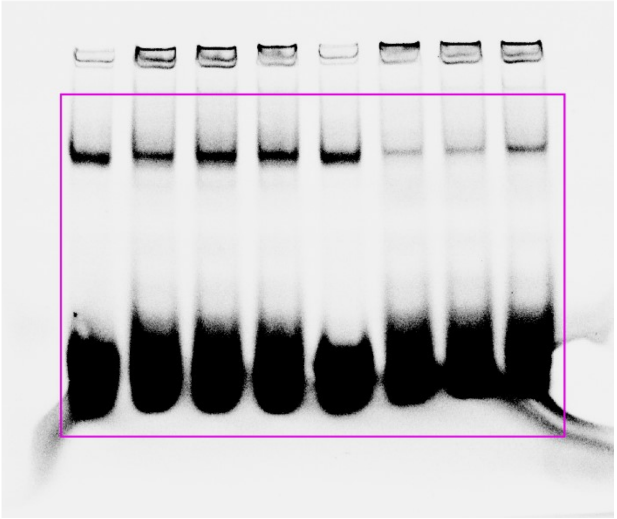

Supplementary Figure 15 Original blot images used in Fig. 5

The panels boxed were used in Fig. 5c-e. The size (kDa) of the marker proteins are marked on the left of each image.

Blots used in Supplementary Fig 8b

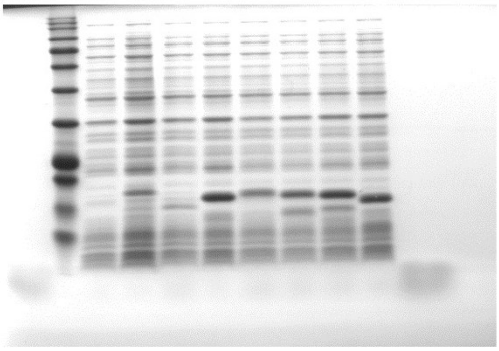

Blots used in Fig 8c

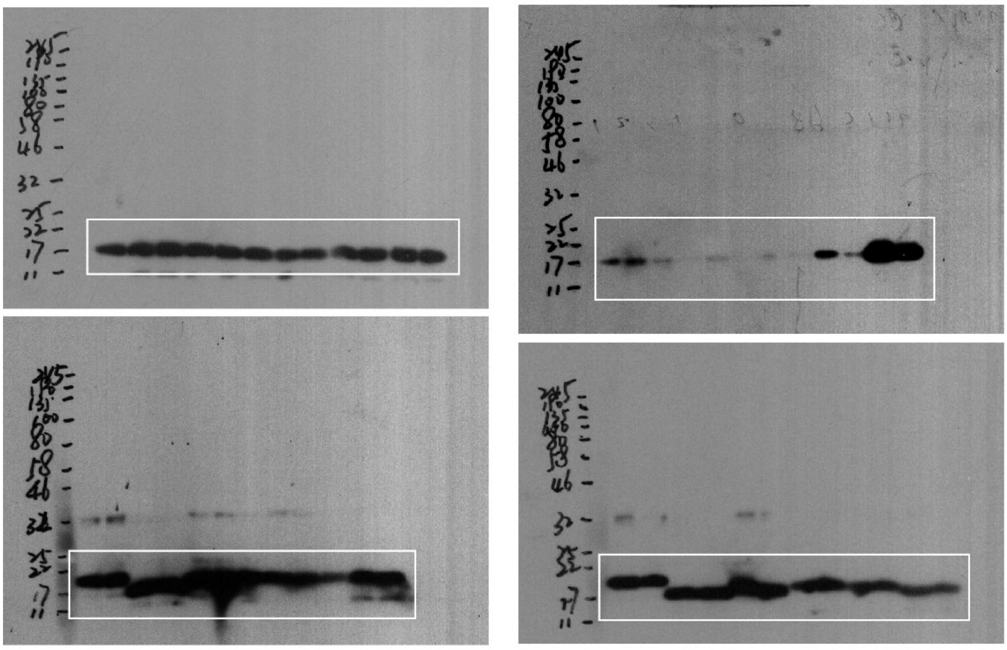

anti-cmyc

anti-HA

Input

IP

Supplementary Figure 16 Original blot images used in Supplementary Fig. 8

The panels boxed were used in Supplementary Fig. 8b,c. The size (kDa) of the marker proteins are marked on the left or right of the images.

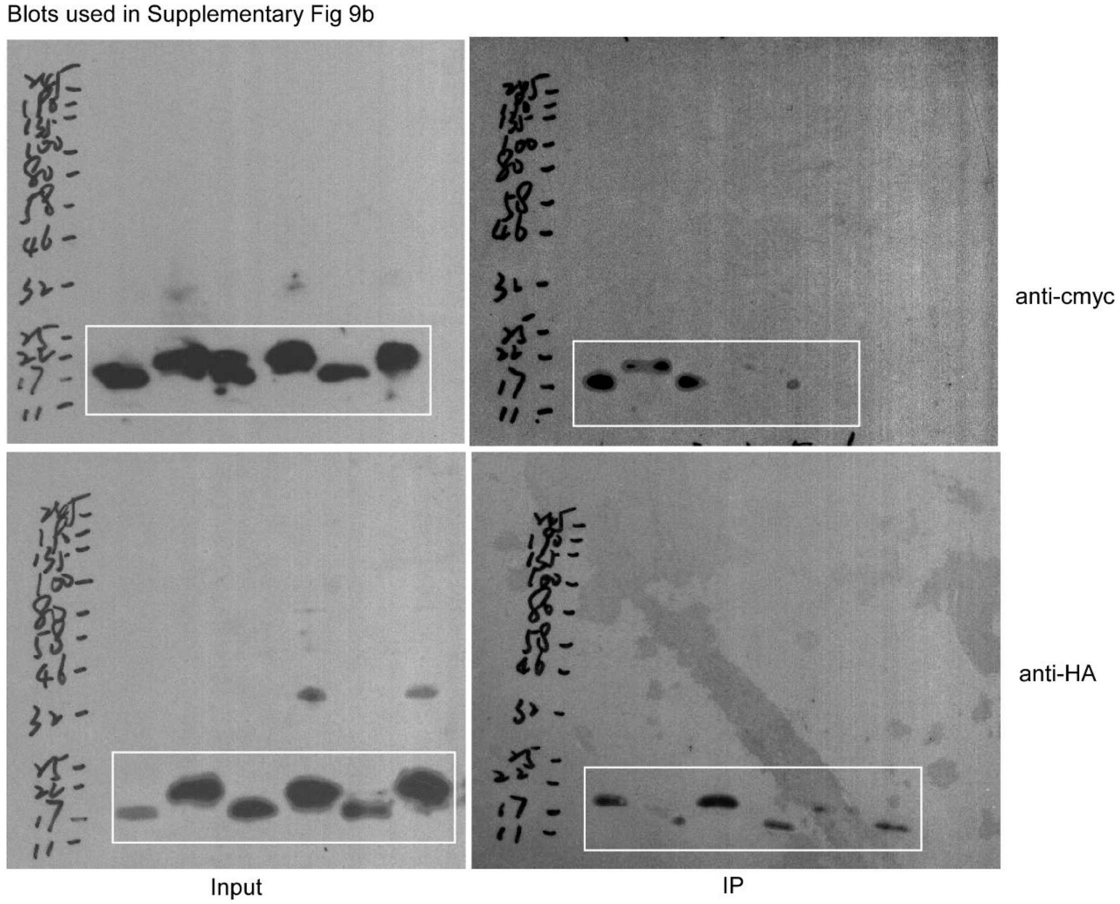

Supplementary Figure 17 Original blot images used in Supplementary Fig. 9

The panels boxed were used in Supplementary Fig. 9b. The size (kDa) of the marker proteins are marked on the left of each image.

Supplementary Table 1 Mapping markers used in this study

Marker	Forward primer sequence (5'-3')	Reverse primer sequence (5'-3')	Marker type	Polymorphism (S.pimp/S.lyc)	Chromosomal Location (SL2.50::chr09)
xps1857	TGCACAAAAGCTAA CGAGTTTC	TGTTGCCATTACCT CTTTTCC	Indel	187/160 bp	2,003,568
xps1858	TCTGACCACAAATA GCAAACTCA	TTGATAAAAATCAT GGTGGAGTG	Indel	176/130	2,947,322
xps1863	CCAAGTTTGGTGAA GGCACT	TGCAAATTTTGAAT TTTGATTGA	Indel	155/128	2,224,510
xps1865	CCTTCCAACAACTA CAATGCATAA	AATTTCTTGTCTCTT GATATCTGTACC	Indel	229/202	2,641,373
xps1866	AGTGTGTATCTGAT AGAGGTGGAGTG	CCCGAGTTAAGATC GTATAATTTTT	Indel	100//74	3,106,484
xps1867	AACAAAAATAAGA CATTTTGCTTCA	TTTTGATAAGGGTA TCGACATTTT	Indel	202/184	3,234,723
xps1869	AACGCCTTGAAAGA GTAAAGACA	TTGCATTTACAACC ACATCAA	Indel	173/151	3,001,915
xps1870	CCCTTTAAACATTT TGTCACACAT	TTCTGTCCATTTTCC TGCAA	Indel	156/137	3,036,073
xps1882	GGAAACTCAATAAC TCTCCTAAACC	CATGGAAGGCGGCT TGTGTATA	CAPS /EcoRI	301 (192/109)	3,035,176
xps1884	GCATACCCCTCATT TCGATG	TTCTGTTTGCCTCTA GCCTTT	CAPS /SpeI	442 (161/281)	3,011,970
xps1889	AAAAAGCCAAAGA GAACACAAT	GGGGAATCTTGGGA CCTATC	CAPS /SmaI	482 (188/294)	3,081,622
xps1890	GCGAAGAAGTGAT GCACAAA	TCAATTCCGAAGAC AATGAAA	CAPS /NheI	410 (38/372)	3,094,021
xps1891	CACACCCCAAAGGA AAAGAA	GTCCTTAGGCCCAA CACAAA	CAPS /BstZ17I	299 (216/84)	3,027,610
xps1892	GGATGATCTAGGTG GACCATAA	GAAAGCCCTGAACC TTTCCT	CAPS /SacI	315 (81/234)	3,060,459
xps1894	AAATCGGTTAACGA TCAATTTCA	AATGCATGCTTTTT GGAACC	CAPS /ScaI	298 (20/268)	3,028,684
xps1898	TGCTCTATGGTGCG AAAATG	AAGGCATCAAGAG GATGCAC	CAPS /MfeI	268 (114/154)	3,044,052
xps1900	ATGAAAATGAGAAT TATACGAGAAAAT	TATGGGCTATGGGG TTGAGA	CAPS /SnaBI	322 (193/129)	3,049,405
xps215	GCCTTTCTTCCAGG ATGCTA	CCCATTTTCCTTCTT CCTAGA	Indel	275/225	3,490,540

Supplementary Table 2 Primers used in this study

Gene	Forward primer sequence (5'-3')	Reverse primer sequence (5'-3')	Description	Size (bp) or position
**RACE primers for DTM cDNA cloning**				
xp3171	AGGCTAAGGAAAAAGGCAGAGT		3' RACE	
xp3172	TATCACAAGCTGGTCCTAGCAA		3' RACE	
xp3173	CAATGACTAGATTGGACCCCTTC		5' RACE	
xp3174	TTCCTCCACCATTGTTGTTG		5' RACE	
xp3171	AGGCTAAGGAAAAAGGCAGAGT		3' RACE	
**Gene editing by CRISPR/Cas9**				
DTM/Solyc09g009620	GATTGGATTTTGATGAAGCATTCT	AAACAGAATGCTTCATCAAAATCC	sgRNA1	236-255
DTM/Solyc09g009620	GATTGGGCTGAAAATGAAAGGCTA	AAACTAGCCTTTCATTTTCAGCCC	sgRNA2	48-67
DTL/Solyc11g007100	GATTGAAAGCTCAACGTCTGAACC	AAACGGTTCAGACGTTGAGCTTTC	sgRNA1	71-90
DTL/Solyc11g007100	GATTGAATAATCTGACAGTTACGC	AAACGCGTAACTGTCAGATTATTC	sgRNA2	30-49
SlREV/Solyc11g069470	GATTGAGCATAGGGAGAGTAGTAG	AAACCTACTACTCTCCCTATGCTC	sgRNA	20-39
xp4115	GTGGTACCCATTCGGAGTTTTTGTATCTTGTTTC	ACGAATTCGCCATTTGTCTGCAGAATTGGC	Cloning sgRNA module into psgR-Cas9-At for constructing 2xsgRNA-Cas9	
DTM/Solyc09g009620	CCCTTTAAACATTTTGTCACACAT	AATATTTAACACCCCCAAAATAAA	mutation identification and genotyping	514 bp (-146~+345)
DTL/Solyc11g007100	CTCAAGGTACTGACCCCCTCT	CTGCAGTTCCAGTGCCTGACGTTTGT	mutation identification and genotyping	440 bp (-209~+231)
SlREV/Solyc11g069470	GCTGCTAAGGAAGTAGTTTCAGG	CTCAATTGTGTGGCCAAGTG	mutation identification and genotyping	3669 bp (-91~+3559)
CAS9	CCCAAGAGGAACAGCGATAAG	GGTCGATGGTGGTGTCAAAG	CAS9-specific primers for genotyping	
**Yeast Two Hybrid and DTM mutagenesis**				
DTM/mDTM	GAATTCATGGACAGAATTAACTCAAAG	GTCGACCTTTTTCTTGGATTTTGATGA	To clone into pBD-GAL4 Cam	264 bp (1~264)
DTL/Solyc11g007100	GTCGACTCATGGAAAAAGAGAATTCA	CTGCAGTTCCAGTGCCTGACGTTTGT	To clone into pBD-GAL4 Cam	231bp (1~231)
SlREV/Solyc11g069470	GAATTCATGGCTATGGTGGCTCAACAG	CTGCAGCACGAATGACCAGTTGATAAA	To clone into pAD-GAL4-2.1, full-length	2523 bp (1~2523)
SlREV-N	GGGGATCCATGGCTATGGTGGCTCAACAG	TTTCTCGAGTTAAGCATAATCAGGAACATCATAAGGATATACACTTTGCAATTGTTGCCG	To clone into pAD-GAL4-2.1 for Y2H assay; Also used for amplifying *SlREVN* cDNA (N-terminal) to be expressed in *E. coli*; HA-tag sequence added to the reverse primer	1-396 bp (1-132aa)
SlHB15A/Solyc03g120910	GAATTCATGGCTTCCTGCAAGGATGGT	GTCGACGACAAATGACCAGTTGACAAAC	To clone into pAD-GAL4-2.1, full-length	2508 bp (1~+2508)
SlHB15A-N	GGGGATCCATGGCTTCCTGCAAGGATG	TTTCTCGAGTTAAGCATAATCAGGAACATCATAAGGATAACAACTGGTATCTTTCGTAGCAAGT	To clone into pAD-GAL4-2.1 for Y2H assay; Also used for amplifying *SlHB15AN* cDNA (N-terminal) to be expressed in *E. coli*; HA-tag sequence added to the reverse primer	1-396 bp (1-132aa)
SlPHB/Solyc02g024070	GAATTCATGGCCTTGTGTTTACAGAGAG	CTCGAGAACAAAAGACCAGTTTATGAAC	To clone into pAD-GAL4-2.1, full-length	2553 bp (1~2553)
SlPHB-N	GGGGATCCATGGCCTTGTGTTTACAGAGAGG	TTTCTCGAGTTAAGCATAATCAGGAACATCATAAGGATACGTGCTGCTAACAGTATTTAT	To clone into pAD-GAL4-2.1 for Y2H assay; Also used for amplifying *SlPHBN*	1-396 bp (1-132aa)

		TTGT	cDNA (N-terminal) to be expressed in *E. coli*; HA-tag sequence added to the reverse primer	
SlHB15B/Solyc12g044410	GAATTCATGTCAATGTCCTGCAAGGAT	GTCGACAACAAACGACCAATTCACAA	To clone into pAD-GAL4-2.1, full-length	2511 bp (1~2511)
SlHB15B-N	GGGGATCCATGTCAATGTCCTGCAAGGATG	TTTCTCGAGTTAAGCATAATCAGGAACATCATAAGGATAACAGCTGGTGTCTTTCGAAGC	To clone into pAD-GAL4-2.1 for Y2H assay; Also used for amplifying *SlHB15BN* cDNA (N-terminal) to be expressed in *E. coli*; HA-tag sequence added to the reverse primer	1-396 bp (1-132aa)
SlPHV/Solyc02g069830	GAATTCATGGATAGTAGCAAGTATGTGA	CTCGAGAATAAAAGACCAGTTTGTGAAC	To clone into pAD-GAL4-2.1, full-length	2502 bp (1~2502)
SlPHV-N	GGGGATCCATGGATAGTAGCAAGTATGTGAGGTA	TTTCTCGAGTTAAGCATAATCAGGAACATCATAAGGATAACTCACGACCACAGACTCACAG	To clone into pAD-GAL4-2.1 for Y2H assay; Also used for amplifying *SlPHVN* cDNA (N-terminal) to be expressed in *E. coli*; HA-tag sequence added to the reverse primer	1-381 bp (1-127aa)
SlHB8/Solyc08g066500	GGATCCATGATGGCTGTGACATCAAGC	CTCGAGGACAAAAGACCAATTGATAAAC	To clone into pAD-GAL4-2.1, full-length	2520 bp (1~2520)
SlHB8-N	GGGGATCCATGATGGCTGTGACATCAAGC	TTTCTCGAGTTAAGCATAATCAGGAACATCATAAGGATAGTTGTCTGTGGTGGCTAAGGC	To clone into pAD-GAL4-2.1 for Y2H assay; Also used for amplifying *SlHB8N* cDNA (N-terminal) to be expressed in *E. coli*; HA-tag sequence added to the reverse primer	1-396 bp (1-132aa)
DTM_L1A	AATTGCTACATAATGGCTGAAAA	TTGCAAGTAGGCCTTTGAGTT	To clone into pBD-GAL4 Cam	264 bp (1~264)
DTM_I15A	ATTGCTACGCAATGGCTGAAAA	TTTGCAAGTAGAGCTTTGAGTTAAT	To clone into pBD-GAL4 Cam	264 bp (1~264)
DTM_L22A	ATGAAAGGGCAAGGAAAAAGG	TTTCAGCCATTATGTAGCAATTTTG	To clone into pBD-GAL4 Cam	264 bp (1~264)
DTM_L29A	GCAGAGTTAGCGAATCAAGAAAAT	CTTTTTCCTTAGCCTTTCATTTTC	To clone into pBD-GAL4 Cam	264 bp (1~264)
DTM_L36A	AGAAAATCAACAAGCTTTGAGTGA	TGATTCAATAACTCTGCCTTTTT	To clone into pBD-GAL4 Cam	264 bp (1~264)
***In situ* hybridization**				
DTM/Solyc09g009620	TAATACGACTCACTATAGGGCGAAATGGACAGAATTAACTCAAAG	GTCGACCTTTTTCTTGGATTTTGATGA	Preparing DNA templates to synthesize sense DTM probe	291 bp (1~291)
DTM/Solyc09g009620	GAATTCATGGACAGAATTAACTCAAAG	TAATACGACTCACTATAGGGCGAACTTTTTCTTGGATTTTGATGA	Preparing DNA templates to synthesize antisense DTM probe	291 bp (1~291)
SlREV/Solyc11g069470	TAATACGACTCACTATAGGGCGAACTTTCTCCAGGCTCACCAGA	TCTTCAGACGACGCAAACAC	Preparing DNA templates to synthesize sense SlREV probe	416 bp (2059~2474)
SlREV/Solyc11g069470	CTTTCTCCAGGCTCACCAGA	TAATACGACTCACTATAGGGCGAATCTTCAGACGACGCAAACAC	Preparing DNA templates to synthesize antisense SlREV probe	416 bp (2059~2474)
SlWUS/Solyc02g083950	TAATACGACTCACTATAGGGCGAATCAGAACCATAAAGCTCGTGA	GGCACATACCAAATTAGATTAGTACT	Preparing DNA templates to synthesize sense SlWUS probe	654 bp (228~881)
SlWUS/Solyc02g083950	TCAGAACCATAAAGCTCGTGA	TAATACGACTCACTATAGGGCGAAGGCACATACCAAATTAGATTAGTACT	Preparing DNA templates to synthesize antisense SlWUS probe	654 bp (228~881)
SlCLV3/Solyc	TAATACGACTCACTA	GGGCCAAAAACAACA	Preparing DNA	349 bp

11g071380	TAGGGCGAAAATCTC TTTGTCTTGCTGATCT GT	AAAAC	templates to synthesize sense SlCLV3 probe	(28~376)
SlCLV3/Solyc 11g071380	AATCTCTTTGTCTTGC TGATCTGT	TAATACGACTCACTA TAGGGCGAA GGGCCAAAAACAACA AAAAC	Preparing DNA templates to synthesize antisense SlCLV3 probe	349 bp (28-376)
SlSTM/Solyc0 2g081120	TAATACGACTCACTA TAGGGCGAATGGTGG ATTGGTGGCTTAGA	TGTACTACTACATGC ACACAAGT	Preparing DNA templates to synthesize sense SlSTM probe	322 bp (818~1139)
SlSTM/Solyc0 2g081120	TGGTGGATTGGTGGC TTAGA	TAATACGACTCACTA TAGGGCGAATGTACT ACTACATGCACACAA GT	Preparing DNA templates to synthesize antisense SlSTM probe	322 bp (818~1139)
**qRT-PCR**				
SleIF4α6	CAGCTTTTGCCACCA AAAAT	TCTGATCCATGTCTCC GTGA	reference gene	350 bp
DTM/Solyc09 g009620	GAATTCATGGACAGA ATTAACTCAAAG	GTCGACCTTTTTCTTG GATTTTGATGA		267 bp (1~267)
SlREV/Solyc 11g069470	CTAAAGAATCTCTGG CAACACC	TTGATAAACGAAAAG GCCAAA		338 bp (2173~2510)
SlWUS/Solyc 02g083950	TCTCCAGCAACTTAC CCTTTTC	TCCAAATAGCTTGGC ACATAC		326 bp (571~896)
SlCLV3/Soly c11g071380	AATCTCTTTGTCTTGC TGATCTGT	GGGCCAAAAACAACA AAAAC		349 bp (28~376)
SlSTM/Solyc 02g081120	TGGTGGATTGGTGGC TTAGA	TGTACTACTACATGC ACACAAGT		322 bp (818~1139)
**Pulldown assay**				
DTM/Solyc09 g009620	B–DTM-F: GGATCC*ATG*GACAGA ATTAACTCAAAG	X-cmyc-DTM-R: CTCGAGTTAAAGATC TTCTTCAGAAATAAG TTTTTGTTCCTTTTTC TTGGATTTTGATGA	cmyc sequence was added to the reverse primer	264 bp (1~264)
SlREV/Solyc 11g069470	B-69470-F: GGATCC*ATG*GCTATG GTGGCTCAACAG	X-HA-69470-R: CTCGAGTTAAGCATA ATCAGGAACATCATA AGGATACACGAATGA CCAGTTGATAAA	HA-tag sequence added to the reverse primer	2523 bp (1~2523)
SlPHV/Solyc 02g069830	B-069830-F: GGATCC*ATG*GATAGT AGCAAGTATGTGA	X-HA-069830-R: CTCGAGTTAAGCATA ATCAGGAACATCATA AGGATAAATAAAAGA CCAGTTTGTGAAC	HA-tag sequence added to the reverse primer	2502 bp (1~2502)
SlHB8/Solyc0 8g066500	B-066500-F: GGATCC*ATG*ATGGCT GTGACATCAAGC	X-HA-066500-R: CTCGAGTTAAGCATA ATCAGGAACATCATA AGGATAGACAAAAG ACCAATTGATAAAC	HA-tag sequence added to the reverse primer	2520 bp (1~2520)
SlHB15A/Sol yc03g120910	B-120910-F: GGATCC*ATG*GCTTCCT GCAAGGATGGT	H-HA-120910-R: AAGCTTTTAAGCATA ATCAGGAACATCATA AGGATAGACAAATGA CCAGTTGACAAAC	HA-tag sequence added to the reverse primer	2508 bp (1~2508)
SlHB15B/Sol yc12g044410	B-44410-F: GGATCC*ATG*TCAATG TCCTGCAAGGAT	X-HA-44410-R: CTCGAGTTAAGCATA ATCAGGAACATCATA AGGATAAACAAACGA CCAATTCACAA	HA-tag sequence added to the reverse primer	2511 bp (1~2511)
SlPHB/Solyc0 2g024070	B-24070-F: GGATCC*ATG*GCCTTG TGTTTACAGAGAG	X-HA-24070-R: CTCGAGTTAAGCATA ATCAGGAACATCATA AGGATAAACAAAAG ACCAGTTTATGAAC	HA-tag sequence added to the reverse primer	2553bp (1~2553)
DTL/Solyc11 g007100	DTL-BamHI-F: GGATCC*ATG*GAAAAA	DTL-XhoI-CMYC-R: CTCGAGTTAAAGATC	cmyc-tag sequence added to the reverse	231bp (1~231)

	GAGAATTCAGA	TTCTTCAGAAATAAGTTTTTGTTCTTCCAGTGCCTGACGTTTGT	primer	
**Bimolecular fluorescence complementation (BiFC) assay**				
DTM/DTM_L8F	GGTACCATGGACAGAATTAACTCAAAG	GTCGACCTTTTTCTTGGATTTTGATGA	To clone into pJW771	264 bp (1~264)
SlREV/Solyc11g069470	GGTACCATGGCTATGGTGGCTCAACAGC	CTGCAGTTACACGAATGACCAGTTGATAA	To clone into pJW772	2526 bp (1~2526)
SlHB15B/Solyc12g044410	CCGGGATCCAATGTCAATGTCCTGCAAGGAT	CAGGTCGACTTAAACAAACGACCAATTCAC	To clone into pJW772	2514 bp (1~2514)
SlHB15A/Solyc03g120910	AAAGGTACCATGGCTTCCTGCAAGGATGGT	AACGTCGACTTAGACAAATGACCAGTTGAC	To clone into pJW772	2511 bp (1~2511)
SlPHB/Solyc02g024070	GTAGGTACCATGGCCTTGTGTTTACAGAGA	AACGGATCCTTAAACAAAAGACCAGTTTAT	To clone into pJW772	2556 bp (1~2556)
SlPHV/Solyc02g069830	GAGGGTACCATGGATAGTAGCAAGTATGTG	AACGGATCCTTAAATAAAAGACCAGTTTGT	To clone into pJW772	2505 bp (1~2505)
SlHB8/Solyc08g066500	GTGGGTACCATGATGGCTGTGACATCAAGC	ATAGGATCCTCAGACAAAAGACCAATTGAT	To clone into pJW772	2523 bp (1~2523)
**EMSA assay**				
DTM/Solyc09g009620	DTM-invitro-F: GCGATCGCTATTTAGGTGACACTATAGAACAGACCACC*ATG*GACAGAATTAACTCAAAG	DTM-invitro-R: GTTTAAACTTAAAGATCTTCTTCAGAAATA	Preparing DNA template for synthesizing protein in TnT™ SP6 High-Yield Wheat Germ Protein Expression System	264 bp (1~264)
SlREV/Solyc11g069470	E-REV-KOZAK-F: GGGAATTCGCCACC*ATG*GCT*ATG*GTGGCTCAACAG	K-REV-HA-N-R: GGGGTACCTTAAGCATAATCAGGAACATCATAAGGATATGTGGGAGCATATATCTGGGTA	Cloning of SlREV (1-265aa) into TNT expression vector	795 bp (1~795)
